# Multivariable machine learning prediction of risky alcohol use in contemporary youth

**DOI:** 10.1111/add.70145

**Published:** 2025-07-16

**Authors:** Lucinda Grummitt, Rachel Visontay, Philip Clare, Tim Slade, Louise Birrell

**Affiliations:** ^1^ The Matilda Centre for Research in Mental Health and Substance Use The University of Sydney Sydney Australia; ^2^ Prevention Research Collaboration, School of Public Health The University of Sydney Sydney Australia; ^3^ National Drug and Alcohol Research Centre UNSW Sydney Sydney Australia; ^4^ Charles Perkins Centre The University of Sydney Sydney Australia

**Keywords:** adolescence, alcohol, cohort study, machine learning, prediction, prevention

## Abstract

**Background and aims:**

Risky alcohol use in young adulthood is a significant public health concern. Understanding the predictors of risky drinking during this period is essential for prevention. This study aimed to measure the predictive accuracy of ensemble machine learning and identify the most important predictors of risky alcohol use in early adulthood.

**Design and setting:**

Secondary analysis of the Longitudinal Study of Australian Children, an Australian national longitudinal cohort study.

**Participants:**

A total of 4983 children, aged 4–5 years in 2004 (Wave 1), followed up for eight waves (to age 18/19 in 2018).

**Measurements:**

Risky alcohol use was measured at age 18 and defined as more than 10 standard drinks per week, as per Australian National guidelines. Predictors from multiple domains—sociodemographic, adolescent substance use, adolescent mental health and behaviours, parental mental health and substance use, school factors, peer influences, parenting practices and parental stress—were included, measured from Wave 1 to 7. The SuperLearner package in R was used to test a series of models [regularised regression (LASSO, ridge and elastic net), random forest and kernel support vector machine (SVM)] using nested 10‐fold cross‐validation to identify the overall predictive ability of the model (measured by area under the curve; AUC) and the most important predictors of risky alcohol use across childhood and adolescence. Predictor importance was derived by normalising algorithm‐specific scores per fold, weighting them by SuperLearner coefficients and aggregating across folds to rank predictors by mean weighted importance on a scale of 0 to 1 (higher scores indicating greater importance).

**Findings:**

The ensemble model showed good prediction on the test set, with an AUC of 0.792, a slight improvement over any single algorithm (AUC = 0.783 for the best performing individual algorithm). The most important predictors were weekly drinking at the previous wave (mean weighted importance 0.999), lifetime cannabis use (0.446), lifetime parent financial stress (0.420), identifying as female (0.365), identifying as male (0.344; compared with a reference category of gender diverse), lifetime attention deficit hyperactivity disorder (0.248), pre‐natal alcohol exposure (0.248), housing insecurity (0.243), religious involvement (0.238) and parent alcohol use problems (0.215).

**Conclusions:**

An ensemble learning approach appears to have good predictive ability of risky alcohol use among a contemporary cohort of young Australians. It underscores the complex interplay of individual, familial and social factors occurring across childhood and adolescence that influences risky alcohol use in early adulthood.

## INTRODUCTION

Alcohol use is a serious public health concern globally. In 2019, it accounted for over 2.4 million deaths [1] and 5% of the overall burden of disease and injury [2]. For young people age 10 to 24 years, alcohol use is the leading risk factor for disability‐adjusted life years [3]. Accordingly, they have been identified as a priority population for alcohol use prevention, with adolescence and emerging adulthood recognised as critical periods for immediate harm and the aetiology of long‐term health and social consequences [4, 5]. Alcohol use typically begins during adolescence, with escalation in early adulthood. The brain continues to develop throughout adolescence and into early adulthood and heavy substance use during this development can severely impact the brain [6, 7] and further increase susceptibility to alcohol‐related harm and alcohol use disorder [8]. Although many improvements in youth alcohol use in high‐income countries have been observed over the past three decades, such as declining rates of use, delayed initiation of substance use until mid‐late adolescence and decreases in harmful alcohol use [9, 10], there remain a substantial proportion of young people who continue to drink alcohol at harmful levels [11]. For example, the proportion of young Australians age 18 to 24 years who are drinking at levels above the low‐risk guidelines (defined as no more than 10 standard drinks weekly and no more than 4 standard drinks in a single day for adults) [12] has remained stable (~42%) in recent years [13]. Indeed, despite the aforementioned encouraging improvements, the peak age of onset for alcohol use disorder is 19.5 years, with 45% of cases of disorder beginning before the age of 25 years [14]. Efforts to prevent risky alcohol use are critical to reducing the associated immediate and long‐term harms for youth.

There are several approaches that have been shown to be effective in preventing risky alcohol use and associated harms [15, 16, 17, 18]. These prevention programs aim to equip young people with knowledge and provide an opportunity to practice social and emotional skills that play key roles in motivations to consume alcohol at risky levels. While interventions to prevent the development of risky alcohol use can be delivered universally, that is, to all adolescents regardless of pre‐existing risk, selective and targeted prevention approaches that focus on individuals or groups at higher risk can optimise prevention efforts and enable more efficient allocation of resources. Identifying which adolescents should be prioritised to receive such interventions through accurate identification of precursive risk factors is an important goal, enabling earlier and more tailored intervention to those more likely to engage in risky alcohol use in early adulthood.

Existing literature suggests many factors are associated with risky alcohol use in early adulthood. A systematic review by Stone and colleagues [19] revealed that at the individual level, these include personality traits such as impulsivity and sensation seeking, a range of externalising behaviours including conduct problems, antisocial behaviours and hyperactivity. Interpersonal risk factors for alcohol use include heavier peer drinking, a family history of problematic substance use, a lack of social support and family relationships high in conflict [19]. Conversely, strong coping skills, positive relationships with family and friends and associations with peers that value sobriety or moderate drinking were found to protect against alcohol use.

Although this evidence is valuable, studies such as those included in the systematic review by Stone and colleagues [19] generally examine the predictors of risky alcohol use in isolation or look at small sets of predictors (e.g. externalising behaviours). In reality, individuals experience a multitude of risk and protective factors for alcohol use, occurring at the individual, interpersonal, community and structural levels that interact in complex ways across development. Analyses that can incorporate predictors across varied domains and across time into the same model are critical in developing an accurate understanding of which of the multitude of previously identified risk and protective factors are the most important in predicting risky alcohol use in adolescence and early adulthood. In recent years, machine learning methods have become more accessible and useful as powerful tools to identify risk factors and examine relationships between a multitude of factors across the life course in large and complex datasets. These techniques offer an advantage over traditional statistical approaches (e.g. logistic regression) through the ability to handle highly correlated data and complex non‐linear relationships that violate the assumptions of logistic regression, but are often present in studies of human behaviour and health outcomes. By leveraging varied algorithms, researchers can account for interactions between numerous predictors, enabling more accurate predictions and insights [20]. Machine learning also allows for the identification of patterns that may be overlooked by traditional statistical methods, facilitating a more comprehensive understanding of the factors associated with alcohol use and ability to predict which young people may engage in risky alcohol use in the future.

Several studies have examined alcohol use in adolescence or early adulthood using machine learning techniques [21, 22, 23, 24, 25]. Three of these examined predictors of heavy episodic drinking specifically [22, 23, 25], while two investigated the frequency of alcohol use in adolescence [21, 24]. This existing literature demonstrates some consistent themes across diverse methodologies and findings. Predictors were found across multiple domains, with those including personality (particularly sensation seeking), psychopathology (especially externalising behaviours), peer influence and substance use history (age at first drink, other drug use) shown consistently to be important in the prediction of alcohol use. The weight of any single predictor was modest, highlighting the multifaceted nature of alcohol use predictors and the value of combining multiple domains within machine learning frameworks. In general, studies that included neurobiological factors or cognitive functioning found these do not substantially improve prediction above psychosocial predictors [21, 22, 25]. From a methodological perspective, multiple machine learning techniques have been used to capture the complex interplay of predictors, with studies comparing different models showing different results in terms of predictive performance: random forest [23], extreme gradient boosting [24] and elastic net [21, 25].

While these studies have advanced understanding of the predictors of risky alcohol use in adolescence and early adulthood, several gaps in the literature remain. The longest follow‐up period assessed was 4 years. Influences on adolescent alcohol use likely occur across development, from early childhood. Indeed, none of the aforementioned studies include factors related to parenting, for example, parental warmth, discipline, closeness of the parent–child relationship or parental monitoring, which is strongly implicated in adolescent alcohol use [26, 27, 28]. In addition, only one study used ensemble machine learning [25]. Ensemble methods incorporate several machine learning algorithms into a composite model that can enhance predictive performance by leveraging the strengths of different algorithms while compensating for their individual weaknesses. In contrast to comparing the performance of several different algorithms run separately, ensemble methods create a weighted average of multiple models, allowing for an adaptive modelling approach. An important advantage is the balance between bias and variance compared to traditional predictive models like logistic regression or single decision trees. Bias refers to errors introduced when a model is too simplistic, missing key patterns in the data. Variance, on the other hand, reflects errors from a model being overly sensitive to small fluctuations in the training data, leading to predictions that do not generalise well. Traditional predictive models like logistic regression may have high bias, while complex models like deep decision trees can suffer from high variance. Ensemble methods address this trade‐off by combining multiple algorithms to reduce both types of errors.

The current study aimed to address these gaps by (1) examining a large set of predictors across childhood and adolescence in a single model; (2) understanding predictive accuracy of an ensemble machine learning approach for risky alcohol use in early adulthood; and (3) identifying the most important predictors of risky alcohol use. We draw on a large dataset of predictors collected across childhood and adolescence among a contemporary cohort of young Australians and make use of advanced ensemble machine learning techniques [a SuperLearner consisting of regularised regression (LASSO, ridge, elastic net), random forest and kernel support vector machine (SVM)].

## METHODS

This study adheres to the Strengthening the Reporting of Observational studies in Epidemiology (STROBE) reporting guidelines and a checklist can be found in the supplement. The analysis was not pre‐registered and the results should be considered exploratory. The current study used data from the K‐cohort of the Longitudinal Study of Australian Children (LSAC), a national longitudinal cohort study that began data collection in 2004. The child cohort (K cohort) were born between March 1999–February 2000 and age 4 to 5 years at baseline. Follow‐up waves of data collection were conducted every 2 years, with the latest wave of data available from 2020 to 2021 (child age 21–22 years in K cohort). LSAC collected data from parents/carers, the study child and teachers and childcare workers involved with the child. Full details of the methodology have been published elsewhere [29]. The current study drew on data from waves 1 to 8 (child age 4/5 years to 18/19 years) of the K cohort of LSAC as the outcome variable of interest was collected at our timepoint of interest (age 18). We drew on data collected from parent 1 (defined as the child's primary caregiver or the parent who knows the child best) and the study child, as well as a handful of variables for parent 2 (e.g. highest education level). This was done to maximise the amount of data available, as data was more commonly missing for parent 2 across all measures and timepoints. Even at baseline, approximately 14% of the sample did not have an eligible parent 2 informant, and even among those eligible, only 79% responded to the survey. For these reasons, imputation of parent 2 data was not deemed appropriate. Moreover, for some of the sample, parent 2 was not living with the child at the time of the survey, therefore, their influence on and knowledge of, child developmental factors would potentially have introduced complex variability to account for. There were also many variables for which a second parent report was not deemed necessary, for example, children's behaviours, for which the report of the parent closest to the child (parent 1) was deemed sufficient. At baseline (mean child age = 4.17 years), 4983 children were present. Response rates for all timepoints can be found in Table 1, and further information on response rates by informant are available in the LSAC data user guide [29].

**TABLE 1:** Response rates for each wave of the LSAC K cohort used in the current study, adapted from Mohal *et al*. [29]

	Child age (y)	Available (*n*)	Rate of original sample still available (%)	Responding (*n*)	Response rate of original sample (%)	Response rate of available sample (%)
Wave 1	4–5	4983				
Wave 2	6–7	4913	98.6	4464	89.6	90.9
Wave 3	8–9	4829	96.9	4331	86.9	89.7
Wave 4	10–11	4774	95.8	4169	83.7	87.3
Wave 5	12–13	4735	95.0	3956	79.4	83.5
Wave 6	14–15	4395	88.2	3537	71.0	80.5
Wave 7	16–17	4176	83.8	3089	62.0	74.0
Wave 8	18–19	3943	79.1	3037	60.9	77.0

*Note*: Available: excludes those who opted out of the study between waves.

Abbreviation: LSAC, Longitudinal Study of Australian Children.

### Measures

Predictor variables were measured up to and including wave 7 (mean age = 16.5 years, SD = 0.51). The outcome variable was measured at wave 8 (mean age = 18.4 years, SD = 0.50). A theory‐driven approach was used to select predictor variables. The literature was searched for recent systematic reviews of child and adolescent factors associated with risky alcohol use or alcohol use disorder in early adulthood and two were identified [19, 30]. The list of predictors identified and their availability in LSAC are reported in Table S1. Predictors that were available in LSAC, as well as any additional variables that were hypothesised to be related to the outcome variable (e.g. discrimination, parent financial stress and unsupervised time) were included in the analysis (220 variables; Table S2). While, given the sample size, this number of predictors exceeds a traditional rule of thumb for logistic regression (10 events per variable), our analytic approach mitigates this rule through the use of base algorithms that use regularisation, non‐parametric modelling and cross‐validated ensemble learning, which optimises predictive performance while reducing overfitting. These methods are designed to handle high‐dimensional data robustly, rendering the traditional 10 events per variable rule less relevant in this context.

#### Outcome: risky alcohol use

At wave 8 (mean age = 18.4), participants were asked about their use of alcohol. A variable was computed to reflect alcohol use that exceeds the low‐risk guidelines (hereafter referred to as ‘risky alcohol use’), as defined by relevant national guidelines (Australian National Health and Medical Research Council; >10 standard drinks in the past week) [12]. Those who responded they had never had an alcoholic drink, had not drunk alcohol in the past year or past month or had drunk 10 or less drinks in the past week were all coded as not engaging in risky alcohol use.

#### Predictors

Detailed information on the items asked and response information for all 220 variables included in the current analysis can be found in Table S2. We included predictor variables from a range of domains:

- Socio‐demographic (gender, socio‐economic position relative to all families, area‐level socio‐economic advantage and disadvantage, parent education, child religious involvement, number of homes since birth, number of schools attended, neighbourhood liveability);
- Parental mental health (any lifetime mental health disorder, perceived difficulty of life, psychological distress, parental self‐efficacy);
- Parental substance use (alcohol use while pregnant with the study child, alcohol use problems, drug use disorder);
- Parenting factors (reported by parent 1: parental monitoring, parental warmth, parental support, activities with child in the home, activities with child outside the home, angry parenting, consistent parenting; reported by the child: unsupervised time, interest in the child's education, close to mother, close to father);
- Parent stress (financial hardship, stressful life events, perceived support available, domestic violence, parental separation, housing insecurity, frequency of couple arguments, relationship quality between parents);
- Adolescent substance use [early onset of alcohol use (full drink by wave 6, mean age = 14.4), weekly drinking by wave 7 (mean age = 16.5), lifetime cannabis use, lifetime other drug use);
- Study child mental health and behaviours [self‐reported, waves 4–7: hyperactivity, emotional problems, conduct problems, sleep quantity; waves 4–5: perceived social support; waves 5–7: depressive symptoms, anxiety symptoms, general happiness, delinquency; waves 6–7: self‐harm ideation, self‐harm, suicidal ideation, suicide plan, suicide attempt; wave 6: big 5 personality; parent reported, all waves: sleep problems, lifetime attention‐deficit hyperactivity disorder (ADHD)];
- School factors (school belonging, parent‐rated school achievement); and
- Peer factors (peer deviancy, bully victimisation, bully perpetration, peer problems, peer trust, peer communication, perceived discrimination).

In general, we made use of all timepoints for which a variable was measured and entered these as separate predictors into the model, which allows for greater flexibility in understanding whether variables may be more or less important at different timepoints. Notable exceptions were for predictors that reflected an exposure where it was hypothesised that the experience would affect the outcome regardless of timing, for example, housing insecurity, measured at age 12, 14 and 16. In these instances, summary variables were created to reflect any exposure, and these were entered alongside the predictors at each timepoint for the model to decide which variable to include. Our machine learning approach is well‐suited to handle highly correlated data without compromising model performance. SuperLearner combines predictions from multiple algorithms (random forests, elastic net and SVM) using non‐negative least squares weights. This ensemble approach mitigates the impact of collinearity by leveraging the strengths of diverse models, each handling correlated predictors differently. For example, random forests are inherently robust to collinearity because they use decision trees, which select one predictor at a time for splitting based on information gain. The elastic net algorithm incorporates both L1 (LASSO) and L2 (ridge) regularisation, explicitly addressing collinearity. L1 regularisation promotes sparsity by shrinking coefficients of redundant predictors to zero, while L2 regularisation stabilises estimates for correlated predictors by distributing weights among them. SVMs with a radial basis function kernel transform predictors into a higher‐dimensional space, where linear correlations in the original space are less problematic. This kernel mapping allows ksvm to focus on non‐linear patterns, reducing the impact of collinearity on classification performance. By weighting algorithms based on cross‐validated performance, SuperLearner reduces reliance on any single model that might be sensitive to collinearity.

### Missing data

Attrition across the 14 years of data collection involved in the current study is shown in Table 1. LSAC has investigated patterns of non‐response extensively, and further information is published elsewhere [31], and also reported in the Supporting information. In addition, the variables to be included in the current analysis were inspected for missingness. The average percent of missing data across all variables was 27%, and no predictors were included where more than 50% of participants had missing responses. Missing data was handled through k‐nearest neighbour (KNN) imputation using the VIM package in R. While preferable to use multiple imputation rather than single imputation of KNN, SuperLearner does not natively handle multiply imputed datasets. For each missing value in a dataset, KNN identifies the k most similar observations, or ‘neighbours,’ (k = 5 in the current study). The missing value is then imputed using a weighted average or majority vote from these nearest neighbours, depending on whether the variable is continuous or categorical. Full details of the imputation procedure are reported in the Supporting information. All presented analyses pertain to the imputed data, except sample characteristics as shown in Table 2.

**TABLE 2:** Socio‐demographic characteristics of the sample.

	*n* (%)
Sex at birth *n* = 4983	
Male	2536 (50.9)
Female	2447 (49.1)
Gender *n* = 2655	
Cisgender male	1321 (49.8)
Cisgender female	1298 (48.9)
Genderqueer/transgender/other/conflicting gender	36 (1.4)
Highest qualification of parents (*n* = 4221)	
Year 12 or lower	543 (12.9)
Diploma/certificate/other	1878 (44.5)
University degree	1800 (42.6)

### Analyses

In pursuit of aim 2 (understanding predictive accuracy of an ensemble machine learning approach for risky alcohol use), we used the ‘SuperLearner’ package in R [32]. SuperLearner implements a machine learning ensemble method that combines multiple prediction algorithms to improve the accuracy and robustness of predictive models [33]. SuperLearner first trains the base algorithms, then uses cross‐validated predictions from these algorithms to derive an optimal weighted combination, known as SuperLearner. This method effectively mitigates the risk of overfitting and enhances predictive performance by leveraging the strengths of different algorithms while compensating for their individual weaknesses. SuperLearner provides coefficients that range from 0 to 1 and reflect how much weight SuperLearner puts on that model in the weighted average. Higher scores indicate the ensemble is weighting that algorithm more highly.

Within SuperLearner we ran regularised regression (Friedman *et al*.) [34], random forest (Wright and Ziegler) [35] and kernel SVM (Karatzoglou *et al*.) [36]. Each of these machine learning algorithms has a set of underlying parameters used to define features of the prediction model (hyperparameters). To optimise model performance, we used a nested cross‐validation framework with hyperparameter tuning. The dataset was split into 15 outer folds (5‐fold cross‐validation repeated 3 times) to evaluate model performance, while inner 10‐fold cross‐validation tuned hyperparameters for three base learners: random forest (ranger), elastic net regression (glmnet) and SVM with radial basis kernel (ksvm). Further details of the hyperparameter tuning are presented in the Supporting information. The SuperLearner combined these learners using non‐negative least squares weighting. The area under the curve (AUC) was calculated to assess predictive performance. AUC was calculated for each outer fold, using SuperLearner predictions and true outcomes, then averaging AUC values across the 15 folds to assess overall predictive accuracy. AUC measures how well the model distinguishes between individuals with and without risky alcohol use. AUC ranges from 0 to 1: a value of 0.5 indicates random guessing (no predictive ability), 0.7 to 0.8 suggests moderate accuracy, 0.8 to 0.9 indicates strong accuracy and above 0.9 is exceptional.

For aim 3 (identifying the most important predictors), feature importance was derived by normalising scores from the included algorithms per fold to 0 to 1, (with 1 indicating high importance and 0 indicating no importance) before weighting by SuperLearner coefficients. These were aggregated across folds to compute the mean and SD of weighted importance and ranked in descending order to identify the most influential predictors.

We additionally ran a sensitivity analysis removing previous substance use in adolescence (weekly drinking at age 16, cannabis use and other drug use). All analyses were performed in R version 4.4.1. R code for these analyses is available at: https://github.com/LucyGrummitt/LSAC_alc_ML.

## RESULTS

Table 2 shows the socio‐demographic characteristics of the sample. Almost all (92%) have consumed alcohol by wave 8, and risky drinking was endorsed by 9% (*n* = 449) of the sample.

Averaging across folds, glmnet emerged as the most effective individual algorithm (coefficient = 0.461), followed by ksvm (coefficient = 0.399) and random forest (coefficient = 0.140). Weights per fold of the cross‐validated SuperLearner are presented in Table S3. Model performance yielded a mean AUC of 0.792 across the 15 folds, which reflects moderate to strong accuracy. This suggests that the model has a reasonable ability to distinguish between individuals who engage in risky alcohol use and those who do not. Although SuperLearner outperformed individual algorithms, the difference was small (Table 3).

**TABLE 3:** Average performance across folds for SuperLearner and base algorithms.

Model	Mean AUC	SD AUC
SuperLearner	0.79224786	0.01474361
Ranger	0.75729407	0.02682725
GLMNET	0.78307302	0.01361559
KSVM	0.77489798	0.0204515

Abbreviation: AUC, area under the curve; SD, standard deviation.

Feature importance, weighted by SuperLearner coefficients and aggregated across folds for the top 20 most important features are presented in Table 4. The most important predictors included weekly drinking at the previous wave (wave 6), lifetime cannabis use, lifetime parental financial stress, being female and being male compared to a reference group of gender diverse. Feature importance for all 220 predictors is reported in Table S4.

**TABLE 4:** Feature importance, weighted by SuperLearner coefficients and aggregated across folds for the top 20 predictors.

Feature (age in years)	Mean weighted importance	SD weighted importance
Weekly drinking (16)	0.99925097	0.002901
Lifetime cannabis use	0.44625379	0.05452562
Financial stress	0.4203031	0.03705029
Female	0.36512711	0.08061375
Male	0.3442192	0.05093263
ADHD	0.24809721	0.05555495
Pre‐natal alcohol exposure	0.24807747	0.04023639
Housing insecurity	0.24328065	0.05270023
Religious involvement	0.23832599	0.04286569
Parent 1 alcohol use problem	0.21496378	0.04816134
Bully victimisation (16)	0.19304537	0.04394482
Anxiety (16)	0.1927689	0.04903178
Moral peers (16)	0.18138694	0.05219186
Neuroticism (16)	0.17994661	0.03000665
Family SES (4)	0.17742442	0.04458989
Lifetime other drug use	0.174376	0.0355995
Lower parental monitoring (14)	0.16979163	0.05638454
Parent 1 self‐efficacy (12)	0.16660787	0.04208602
Moral peers (14)	0.16515154	0.04821889

*Note*: Ages are provided in brackets where the predictor was selected at a specific age by SuperLearner.

Abbreviations: ADHD, attention‐deficit hyperactivity disorder; SD, standard deviation; SES, socio‐economic status.

Results of the sensitivity analysis removing predictors capturing previous substance use in adolescence (weekly drinking at age 16, cannabis use and other drug use) are reported in the Supporting information (Tables S5–S7). As expected, the overall AUC was slightly reduced (0.771 compared to 0.792), but generally results were similar. The most important predictors were parent financial stress, gender identity, religious involvement and parent alcohol use problem.

## DISCUSSION

In the longest follow‐up period of any machine learning study to predict alcohol use, the current study provides insight into the utility of ensemble machine learning to predict risky alcohol use from a broad range of risk and protective factors from early childhood through adolescence. Overall, SuperLearner showed good performance in predicting risky alcohol use on unseen data, with an AUC of 0.792. The ensemble approach outperformed any single algorithm and weighted the regularised regression highest, followed by kernel SVM and random forest.

By including a large and diverse range of predictors into a single modelling approach, the current study was able to compare the importance of these predictors in predicting risky alcohol use at age 18. The 10 most important predictors were weekly drinking at the previous wave (wave 6), lifetime cannabis use, lifetime parent financial stress, identifying as female, identifying as male (compared to a reference category of gender diverse), lifetime ADHD, pre‐natal alcohol exposure, housing insecurity, religious involvement and parent alcohol use problems. This highlights the importance of applying a life course perspective to understanding the predictors of risky alcohol use in early adulthood. Using the most important features in SuperLearner to improve prediction of risky alcohol use may enable earlier and more efficient alcohol prevention programs and messaging by targeting resources to those most at risk.

Although the current study can indicate life course factors that help to predict risky alcohol use, it cannot identify whether any of the variables are causally related to risky alcohol use. There is a growing push to integrate concepts of causal reasoning into machine learning to improve the value that these models can provide to social scientists [37, 38]. Therefore, an important extension of this study is to identify which predictors may be causal and estimate their causal effects on risky alcohol use. Targeted maximum likelihood estimation (TMLE) [39] and target trial emulation (TTE) [40] are advanced methods designed for causal inference, offering robust approaches to address confounding and other biases inherent in observational data. TMLE combines machine learning with causal modelling to produce doubly robust estimates, ensuring consistency even when certain modelling assumptions are violated. TTE, on the other hand, mimics the design and analysis of a randomised controlled trial within observational datasets, providing a framework for estimating causal effects by explicitly defining treatment interventions and comparison groups. These methods would allow for a rigorous examination of whether predictors identified in the current analysis are causal and quantify their impact on risky alcohol use.

This study has several important limitations that should be considered when interpreting the results. First, this study was not pre‐registered and, therefore, should be considered exploratory. Second, our outcome variable was self‐reported based on participant recall of their alcohol consumption over the past week. This may have led participants to over‐ or under‐report their actual drinking, and in our sample only 9% were categorised as engaging in risky drinking at age 18. There is also the potential that the past week did not reflect a typical week of drinking for that participant. Unfortunately, LSAC only captured the number of alcohol drinks consumed over the past week, therefore, we are unable to determine participants level of drinking over other time periods. Third, the quality of our predictions depends on the quality and availability of the measures included in the LSAC survey. Some factors identified in the systematic review were not included in LSAC, most notably, a measure of childhood traumatic events and contextual factors such as alcohol outlet density, laws and taxation, which are known to be strongly associated with alcohol use [41, 42]. Moreover, LSAC was not established to predict alcohol use specifically, and therefore, some of the scales that were included may not be as sensitive in predicting alcohol use as other existing scales. For example, the 10‐item personality questionnaire included in the LSAC is brief, and there are known personality measures not included that show better predictive accuracy for alcohol use [43]. Therefore, it is likely that prediction of risky alcohol use would be improved by using a survey specifically designed to understand risk and protective factors of risky alcohol use. Fourth, the findings are based on a single, national dataset from LSAC. Without external validation, we do not know how well our model would predict risky alcohol use in a completely new sample or whether the same individual predictors would be similarly important. Future research should assess the generalisability of these findings to new and diverse samples. Additionally, while we identified a wide range of key predictors across different ages, this limits the clinical utility of our predictive model in practice. Settings that deliver alcohol use prevention, such as schools, are unlikely to have or be able to collect this breadth of data. Future research would benefit from developing predictive models for risky alcohol use that more closely mirror the type of data that real‐world settings can collect. Of course, given self‐report data on individual attributes may be the most feasible for settings to collect, this would inevitably miss broader parent and social determinants of alcohol use. Therefore, there are trade‐offs to each approach. Another limitation of our study is that our analysis did not account for the fact that we included repeated measures collected over time. Machine learning approaches do not natively account for non‐independence of data through repeated measures, and there is currently no accepted standard for how to deal with this, particularly when combining individual base algorithms that handle predictors differently into an ensemble model. Additionally, while we addressed data missingness using KNN imputation, this is a single imputation approach that does not account for uncertainly in the imputed values, unlike the more robust multiple imputation. While KNN imputation is a common approach in machine learning tasks because of the prioritisation of prediction accuracy and the lower computational intensity, it is possible we did not fully capture the underlying missingness mechanisms in our data. Moreover, while machine learning techniques provide valuable insights, they are ‘black‐box’ models, and the specific pathways underlying the identified relationships are not always interpretable. We tried to overcome this limitation in the current study by using domain‐specific knowledge to drive feature selection and extracting the average feature importance. However, there is still much complexity in the relationships between variables and the algorithms used that is not interpretable or available. This was particularly relevant for understanding the direction of relationships between predictors and outcome, which cannot readily be extracted from SuperLearner. Although SuperLearner did show the best predictive performance with an AUC of 0.792, this was a marginal improvement in prediction compared to the regularised regression algorithm (AUC = 0.783), for which coefficients can be directly interpreted as per traditional regression approaches. Therefore, it is important for researchers to consider the goal of their analyses carefully, and if understanding the nature of relationships is paramount, it may be worthwhile to prioritise individual machine learning algorithms rather than an ensemble approach.

Nonetheless, this study highlights the predictive ability of an ensemble learning approach to the prediction of risky alcohol use among a contemporary cohort of young Australians. It highlights that for those young people who consume alcohol at risky levels there continues to be a complex interplay of individual, familial and social factors occurring across childhood and adolescence that influences risky alcohol use in early adulthood. These findings can inform which young Australians may benefit most from interventions to prevent risky alcohol use, with the hope of ultimately reducing the burden of disease associated with alcohol consumption.

## AUTHOR CONTRIBUTIONS

**Lucinda Grummitt:** Conceptualization (equal); data curation (lead); formal analysis (lead); methodology (lead); project administration (lead); writing—original draft (lead); writing—review and editing (lead). **Rachel Visontay:** Methodology (supporting); validation (supporting); writing—review and editing (equal). **Philip Clare:** Conceptualization (supporting); methodology (supporting); supervision (supporting); writing—review and editing (equal). **Tim Slade:** Conceptualization (supporting); methodology (supporting); supervision (supporting); writing—review and editing (equal). **Louise Birrell:** Conceptualization (equal); funding acquisition (lead); methodology (supporting); project administration (supporting); supervision (lead); writing—review and editing (equal).

## DECLARATION OF INTERESTS

None.

## Supporting information


**Table S1.** Child and adolescent factors associated with young adult alcohol use, as identified by Stone et al. (2012) [1] and Meque et al. (2019) [2].
**Table S2.** Full details of measures used to assess childhood and adolescent risk factors for risky alcohol use at age 18.
**Table S3.** SuperLearner weights and AUD for the ensemble for all 15 folds, as well as the average weighting across all 15 folds.
**Table S4.** Feature importance, normalised and weighted by SuperLearner coefficients and aggregated across folds for all 220 predictors.
**Table S5.** SuperLearner weights for all 15 folds, as well as the average weighting across all 15 folds.
**Table S6.** Average performance across folds for SuperLearner and base algorithms.
**Table S7.** Feature importance, normalised and weighted by SuperLearner coefficients and aggregated across folds for all 217 predictors included in the sensitivity analysis.

## Data Availability

Applications for data access can be submitted via the Growing Up in Australia website: https://growingupinaustralia.gov.au/data-and-documentation/accessing-lsac-data.
