# Supplementary material for: Multivariable machine learning prediction of risky alcohol use in contemporary youth
Source: Addiction. 2025 Jul 16;120(12):2404–12. doi: 10.1111/add.70145 (PMC12586787; doi:10.1111/add.70145)
Supplement: Supplementary file 1 — Table S1. Child and adolescent factors associated with young adult alcohol use, as identified by Stone et al. (2012) [1] and Meque et al. (2019) [2]. Table S2. Full details of measures used to assess childhood and adolescent risk factors for risky alcohol use at age 18. Table S3. SuperLearner weights and AUD for the ensemble for all 15 folds, as well as the average weighting across all 15 folds. Table S4. Feature importance, normalised and weighted by SuperLearner coefficients and aggregated across folds for all 220 predictors. Table S5. SuperLearner weights for all 15 folds, as well as the average weighting across all 15 folds. Table S6. Average performance across folds for SuperLearner and base algorithms. Table S7. Feature importance, normalised and weighted by SuperLearner coefficients and aggregated across folds for all 217 predictors included in the sensitivity analysis. [file ADD-120-2404-s001.docx]

**Supplementary file**

STROBE Statement—Checklist of items that should be included in reports of ***cohort studies***

|  | Item No | Recommendation | Page No |
| --- | --- | --- | --- |
| **Title and abstract** | 1 | (*a*) Indicate the study’s design with a commonly used term in the title or the abstract | 1  2 |
|  |  | (*b*) Provide in the abstract an informative and balanced summary of what was done and what was found |  |
| Introduction | | | |
| Background/rationale | 2 | Explain the scientific background and rationale for the investigation being reported | 4-8 |
| Objectives | 3 | State specific objectives, including any prespecified hypotheses | 8 |
| Methods | | | |
| Study design | 4 | Present key elements of study design early in the paper | 8 |
| Setting | 5 | Describe the setting, locations, and relevant dates, including periods of recruitment, exposure, follow-up, and data collection | 8-9 |
| Participants | 6 | (*a*) Give the eligibility criteria, and the sources and methods of selection of participants. Describe methods of follow-up | 8-9 |
|  |  | (*b*) For matched studies, give matching criteria and number of exposed and unexposed |  |
| Variables | 7 | Clearly define all outcomes, exposures, predictors, potential confounders, and effect modifiers. Give diagnostic criteria, if applicable | 9-11 |
| Data sources/ measurement | 8* | For each variable of interest, give sources of data and details of methods of assessment (measurement). Describe comparability of assessment methods if there is more than one group | Supplement |
| Bias | 9 | Describe any efforts to address potential sources of bias | 12-13 |
| Study size | 10 | Explain how the study size was arrived at | 9 |
| Quantitative variables | 11 | Explain how quantitative variables were handled in the analyses. If applicable, describe which groupings were chosen and why | Supplement |
| Statistical methods | 12 | (*a*) Describe all statistical methods, including those used to control for confounding | 12-14 and supplement |
|  |  | (*b*) Describe any methods used to examine subgroups and interactions |  |
|  |  | (*c*) Explain how missing data were addressed |  |
|  |  | (*d*) If applicable, explain how loss to follow-up was addressed |  |
|  |  | (*e*) Describe any sensitivity analyses |  |
| Results | | |  |
| Participants | 13* | (a) Report numbers of individuals at each stage of study—eg numbers potentially eligible, examined for eligibility, confirmed eligible, included in the study, completing follow-up, and analysed | 9 |
|  |  | (b) Give reasons for non-participation at each stage |  |
|  |  | (c) Consider use of a flow diagram |  |
| Descriptive data | 14* | (a) Give characteristics of study participants (eg demographic, clinical, social) and information on exposures and potential confounders | 15 and supplement |
|  |  | (b) Indicate number of participants with missing data for each variable of interest |  |
|  |  | (c) Summarise follow-up time (eg, average and total amount) |  |
| Outcome data | 15* | Report numbers of outcome events or summary measures over time | 15 |

| Main results | 16 | (*a*) Give unadjusted estimates and, if applicable, confounder-adjusted estimates and their precision (eg, 95% confidence interval). Make clear which confounders were adjusted for and why they were included | 15 |
| --- | --- | --- | --- |
|  |  | (*b*) Report category boundaries when continuous variables were categorized |  |
|  |  | (*c*) If relevant, consider translating estimates of relative risk into absolute risk for a meaningful time period |  |
| Other analyses | 17 | Report other analyses done—eg analyses of subgroups and interactions, and sensitivity analyses | 14 and supplement |
| Discussion | | | |
| Key results | 18 | Summarise key results with reference to study objectives | 16 |
| Limitations | 19 | Discuss limitations of the study, taking into account sources of potential bias or imprecision. Discuss both direction and magnitude of any potential bias | 17-19 |
| Interpretation | 20 | Give a cautious overall interpretation of results considering objectives, limitations, multiplicity of analyses, results from similar studies, and other relevant evidence | 19 |
| Generalisability | 21 | Discuss the generalisability (external validity) of the study results | 19 |
| Other information | | | |
| Funding | 22 | Give the source of funding and the role of the funders for the present study and, if applicable, for the original study on which the present article is based | 1 |

*Give information separately for exposed and unexposed groups.

**Note:** An Explanation and Elaboration article discusses each checklist item and gives methodological background and published examples of transparent reporting. The STROBE checklist is best used in conjunction with this article (freely available on the Web sites of PLoS Medicine at http://www.plosmedicine.org/, Annals of Internal Medicine at http://www.annals.org/, and Epidemiology at http://www.epidem.com/). Information on the STROBE Initiative is available at http://www.strobe-statement.org.

Supplementary Table 1. Child and adolescent factors associated with young adult alcohol use, as identified by Stone et al. (2012)[1] and Meque et al. (2019)[2]

| **Category** | Available in LSAC Waves 1 - 7 | Used in the current study | Notes |
| --- | --- | --- | --- |
| Sex/Gender | Yes | Yes |  |
| Race/ethnicity | No | No | Some proxies / related constructs are available, such as language spoken at home, country of birth, and Aboriginal or Torres Strait Islander status. The latter requires specific ethic approval which was not obtained, and therefore this was not included in the current study. |
| Biological indicators | No | No |  |
| Pre-natal alcohol exposure | Yes | Yes |  |
| Weaning 2 weeks post-birth and fed according to a set schedule rather than on-demand | No | No |  |
| Perinatal obstetric situation | No | No |  |
| Income/socioeconomic status (SES) | Yes | Yes |  |
| Parental education | Yes | Yes |  |
| Parent separation | Yes | Yes |  |
| Family substance use history | Yes | Yes |  |
| Parental psychopathology | Yes | Yes |  |
| Neighborhood instability | Yes | Yes | Neighbourhood liveability, as well as the number of homes lived in since birth as a proxy for low attachment to neighbourhoods |
| Social norms | No | No |  |
| Laws and taxation | No | No |  |
| Availability | No | No |  |
| History of abuse/neglect | No | No |  |
| Parent affection | Yes | Yes |  |
| Parent support | Yes | Yes |  |
| Family conflict | Yes | Yes |  |
| Parental monitoring | Yes | Yes |  |
| Hyperactivity | Yes | Yes |  |
| Antisocial behaviour | Yes | Yes |  |
| Negative emotions / negative affect | Yes | Yes |  |
| Neurobehavioural disinhibition | No | No |  |
| Novelty seeking / sensation seeking | Yes | Yes | Included extraversion and openness |
| Conduct problems | Yes | Yes |  |
| Aggression | Yes | Yes | Captured via delinquency and conduct scales |
| Delinquency / deviancy | Yes | Yes |  |
| Truancy | Yes | Yes | Included as an item in the delinquency scale |
| Depressive symptoms / internalising symptoms | Yes | Yes |  |
| Anxiety | Yes | Yes |  |
| Externalising | Yes | Yes |  |
| Early drinking onset | Yes | Yes |  |
| Drinking frequency | Yes | Yes |  |
| Substance use attitudes and expectancies | No | No |  |
| Illicit drug use | Yes | Yes |  |
| Job status | Yes | No | Given the age of the sample at the last timepoint for predictor measurement (16 years), this was not included. |
| College attendance | No | No |  |
| Peer relations | Yes | Yes |  |
| Belief in conformity or the moral order | No | No |  |
| Religious involvement | Yes | Yes |  |
| Educational factors | Yes | Yes |  |
| Becoming pregnant | Yes | No | Given the age of the sample at the last timepoint for predictor measurement (16 years), this was not included. |
| Marriage | No | No |  |
| Stressful life events | No | No | Included parent stressful life events across all study waves |
| School performance | Yes | Yes |  |
| Bonding to school | Yes | Yes |  |

| Not available to include |
| --- |
| Author decision not to include |
| Included |

Supplementary Table 2. Full details of measures used to assess childhood and adolescent risk factors for risky alcohol use at age 18.

| **Domain** | | | | **Measure** | | **Item response and scale details** | **Coding in current analysis** |  |
| --- | --- | --- | --- | --- | --- | --- | --- | --- |
| **Socio-demographics** | | | |  | |  |  |  |
|  | | Gender | | At Wave 8 (age 18/19), participants were asked: What is your current gender identity? At Wave 1 (child age 4/5), parents were asked the sex of their child (male or female). | | Participants at Wave 8 could indicate male, female, genderqueer/gender non-conforming/ other. Those assigned male at birth and identified as male at Wave 8 were coded as cisgender male; those assigned female at birth and identified as female at Wave 8 were coded as cisgender female. Those whose gender identity conflicted with their sex assigned at birth were coded as transgender/other/ having a conflict with sex assigned at birth. | 2 variables. Dummy variables of cisfemale and cismale were entered into the model; with Genderqueer/ Transgender/ Other/Conflict with Gender serving as the reference category. |  |
|  | | Socio-economic position | | Socio-economic position relative to all families at Wave 1 (age 4/5). Continuous measure that combines parent income, years of education, and occupational prestige. | | Z scores. | 1 variable. Continuous. |  |
|  | | SEIFA | | Area-level index of socio-economic advantage and disadvantage (IRSAD). Consists of census data such as income, education, employment, occupation, housing and family structure to summarise the socio-economic characteristics of an area. | | Each area receives a SEIFA score indicating how relatively advantaged or disadvantaged that area is compared with other areas.  Low scores indicate greater disadvantage and a lack of advantage in general. | 7 variables reflecting IRSAD at each timepoint from Wave 1 – Wave 7. Continuous. |  |
|  | | Parent education | | At Wave 1, both parents reported highest level of school completed and highest post-secondary qualification. | | These were categorised into Year 12 or lower; diploma/certificate/other; university degree, and the highest qualification of the household (maximum of either parent) was computed. | 2 variables. Dummy variables of diploma and university degree, with Year 12 or lower as the reference category. |  |
| **Housing** | | | |  | |  |  |  |
|  | | Homes lived in since birth | | Summary variable at child age 14 reflecting parent-reported number of homes the child had lived in since birth. | | Number. Responses ranged from 1-18. | 1 variable. Continuous. |  |
|  | | Neighbourhood liveability | | At Wave 1-4 and Wave 6, parent 1 was asked: How strongly do you agree or disagree with these statements about your neighbourhood? This is a safe neighbourhood; and There are good parks, playgrounds and play spaces in this neighbourhood. ('Neighbourhood' is as defined by respondent) | | Possible responses for each item were: 1 Strongly agree; 2 Agree; 3 Disagree; 4 Strongly disagree. A mean score across both items was computed, with higher scores indicating worse neighbourhood conditions. | 5 variables; 1 at each timepoint measured. Continuous. |  |
|  | | Housing security | | At Waves 5-7, parent 1 was asked whether they had always had a permanent place to live in the last 2 years. | | 0 No; 1 Yes | 4 variables: 3 binary yes/no at each wave and 1 summary variable reflected any waves of not having always had a permanent place to live. |  |
| **Parent mental health / substance use** | | | | | |  |  |  |
|  | | Alcohol use during pregnancy | | At Wave 1 (age 4/5), the biological mother was asked, “During the pregnancy with the study child, did you drink alcohol?” | | Response options were 1 Yes, occasionally; 2 Yes, most days; 3 No. Children whose mothers reported any alcohol consumption were coded as having prenatal alcohol exposure. | 1 variable. Binary yes/no. |  |
|  | |  | Parent mental disorder | | At Wave 7 (age 16/17), Parent 1 was asked whether they had ever had any of the following conditions, with separate items for depression/anxiety, bipolar, schizophrenia, postnatal depression, other mental illness. | Response options were 1 No, never; 2 Yes, as a child only; 3 Yes, as an adult only; 4 Yes, as a child and as an adult. Parents who responded with options 2-4 to any mental disorder category were coded as having experienced a lifetime mental disorder. | 1 variable. Binary yes/no. | |
|  | |  | Parent problematic alcohol use | | From Waves 1-6, Parent 1 reported on their alcohol use patterns. Problematic alcohol use was defined as having heavy daily alcohol consumption (>4 drinks for men >2 for women) or frequent binge drinking (7+ drinks in a sitting for men 5+ for women 2 to 3 times a month or more often). | If the parent reported problematic alcohol use at two or more waves, that parent was coded as yes to problematic alcohol use. | 1 variable. Binary yes/no. | |
|  | |  | Household drug use problems | | At Waves 5-6, Parent 1 was asked whether they had ever had any of the following conditions, with “Drug Addiction” listed as one possible condition. | Response options were 1 No, never; 2 Yes, as a child only; 3 Yes, as an adult only; 4 Yes, as a child and as an adult. Parents who responded with options 2-4 at either wave were coded as having experienced a drug use problem. | 1 variable. Binary yes/no. | |
|  | |  | Parent 1 psychological distress | | From Waves 1-7, parent 1 completed the Kessler Psychological Distress Scale (K6). | Scores were summed to create a total score, with higher scores reflecting greater psychological distress. | 7 variables. Continuous scores at each timepoint measured. | |
| **Parenting** | | | |  | |  |  |  |
|  | | Unsupervised time | | At child age 12/13 and 14/15, child asked, (Last week) on how many days did you spend an hour or more with no grownups around? | | Response options 0 Never; 1 1 day; 2 2 days; 3 3 days; 4 4 days; 5 Every day. | 2 variables; one at each timepoint. Ordinal. |  |
|  | | Parental warmth | | Assessed from Wave 1-6 (ages 4/5 – 14/15) with six items drawn from the Child Rearing Questionnaire (CRQ)[3]. This was completed by Parent 1 and measured how often they display physical affection, warmth and closeness to the child. | | Response options ranged from 1 Never/Almost never; 2 Rarely; 3 Sometimes; 4 Often; 5 Always/Almost always. The mean score across the 6 items at each wave was computed, with higher scores reflecting greater parental warmth. | 7 variables; one at each timepoint plus a summary variable which was the maximum parental warmth score across all timepoints. Continuous. |  |
|  | | Lower parental monitoring | | At Waves 3-6, Parent 1 was asked the following three questions:  In the course of a day, how often do you know where child is?; How often do you know who child is with when he/she is away from home?; How often does child go out without telling you where s/he will be? | | Response options ranged from 1 Always; 2 Almost always; 3 About half the time; 4 Almost never; 5 Never. The last item was reverse coded and the mean of the three items was calculated, such that higher scores reflected less knowledge of the child’s whereabouts. | 4 variables; one at each timepoint. Continuous. |  |
|  | | Closeness to parent | | From Waves 5-7 (child age 12/13 – 16/17), child was asked two items: how close they feel to their mum, and how close they feel to their dad. | | Responses ranged from 1 Very close; 2 Quite close; 3 Not very close; 4 Not close at all. Scores from 1-2 were coded as close to that parent at that timepoint, scores 3-4 were coded as not close. | 5 variables: close to mother at each timepoint; close to father at 12/13 and 14/15. Binary yes/no. |  |
|  | | Parental support to child | | At Waves 5-6 (child age 14/15 – 16/17), Parent 1 was asked about how often they and the study child talk about: what is going on in his/her life, how things are going with his/her friends, his/her plans for the future, problems he/she is having at school, future jobs he/she might have, what courses he/she should take in school and how these courses will prepare him/her for these future jobs. | | Response options ranged from 1 Never/Almost never; 2 Rarely; 3 Sometimes; 4 Often; 5 Always/Almost always. A mean score for all items was computed at each timepoint, with higher scores reflecting greater parental support to the child. | 2 variables; one at each timepoint. Continuous. |  |
|  | | Parental self-efficacy | | At Waves 1-7, Parent 1 was asked: “Overall, which one of the following statements best describes how you feel about yourself as a parent?” | | 1 Not very good at being a parent; 2 A person who has some trouble being a parent; 3 An average parent; 4 A better than average parent; 5 A very good parent | 7 variables; one at each timepoint. Ordinal. |  |
|  | | Consistent parenting | | At Waves 1-7, Parent 1 was asked the following 5 items: 1-When you give this child an instruction or make a request to do something, how often do you make sure that he/she does it? 2- If you tell this child he/she will get punished if he/she doesn't stop doing something, but he/she keeps doing it, how often will you punish him/her? 3-How often does this child get away with things that you feel should have been punished? 4-How often is this child able to get out of punishment when he/she really sets his/her mind to it? 5-When you discipline this child, how often does he/she ignore the punishment? | | Response options were 1 Never/Almost never; 2 Less than half the time; 3 About half the time; 4 More than half the time; 5 All the time Items 3, 4, and 5 were reverse coded, and the mean of all items was calculated at each timepoint, with higher scores reflecting more consistent parenting. | 7 variables; one at each timepoint. Continuous. |  |
|  | | Home activities index | | At Waves 1-4, Parent 1 was asked variations on the following 7 items: “In the past week, on how many days have you or someone in your family done the following with child?  1-Read to child from a book?  2-Told child a story, not from a book?  3-Drawn pictures or did other art or craft activities with child?  4-Played music, sang songs, danced or did other musical activities with child?  5-Played with toys or games indoors, like board or card games with child?  6-Involved child in everyday activities at home, such as cooking or caring for pets?  7-Played a game outdoors or exercised together like walking, swimming, cycling?”  At Wave 1 -2, and 4, Parent 1 was asked all 7 of these items. At Wave 3, Parent 1 was asked items 2, 6, and 7. | | Response options ranged from: 0 Not in the past week; 1 1 or 2 days; 2 3-5 days; 3 6-7 days. A mean score across all items was computed for each timepoint. | 4 variables; one at each timepoint. Continuous. |  |
|  | | Out of home activities | | At Waves 1-6, Parent 1 was asked: “In the past month say from (nominate date one month ago), has child done any of these things with you or another family member? Gone to a movie or watch a sporting event?  Gone to a playground or a swimming pool?  Attended a religious service, church, temple, synagogue or mosque?  Gone to a live performance for children, like a concert or play/attended a school, cultural or community event/visited a museum or art gallery?  Visited a library?” | | Response options were: 0 No; 1 Yes. A total score was computed reflecting the number of Yes responses. | 6 variables; one at each timepoint. Continuous. |  |
|  | | Angry parenting | | At Waves 1-7, Parent 1 was asked the following: “When parents spend time with their children, sometimes things go well and sometimes they don’t. How often does the following happen...? Of all the times you talk to this child about his/her behaviour, how often is this praise  Of all the times you talk to this child about his/her behaviour, how often is this disapproval  How often are you angry when you punish this child  How often do you feel you are having problems managing this child in general” | | Response options ranged from: 1 Never/Almost never; 2 Less than half the time; 3 About half the time; 4 More than half the time; 5 All the time. The first item was reverse coded, then a mean across all items was computed at each timepoint, for parent 1 and parent 2 separately. | 7 variables: one at each timepoint. Continuous. |  |
| **Parent stress** | | | |  | |  |  |  |
|  | | Parental separation | | At Wave 7 (age 16), parent reported whether their child has a parent living elsewhere. | | Binary yes/no. | 1 variable. Binary. |  |
|  | | Financial hardship | | At Waves 2 to 6, Parent 1 rated how hard it would be for them to raise $2000 in only one week. | | At each wave parents were considered to have difficulty raising the money if they responded “I don’t think I could raise the money”, and able to raise the money if they responded: “I could easily raise the money”, “I could raise the money, but it would involve some sacrifices”, “I would have to do something drastic to raise the money” | 6 variables. Binary yes/no as to whether parent couldn’t raise the money at each timepoint; and one summary variable reflecting any waves of financial hardship. |  |
|  | | Domestic violence | | At Waves 1 to 6, Parent 1 rated how often they have arguments with their partner that end up with people pushing, hitting, kicking or shoving? | | Response options included 1 Never; 2 Rarely; 3 Sometimes; 4 Often; 5 Always. Variables were recoded into a binary variable with all responses above 1-Never coded as experiencing domestic violence at that timepoint. | 7 variables. Binary yes/no for each timepoint, as well as 1 summary score reflecting any waves of domestic violence. |  |
|  | | Parent relationship quality | | At Waves 1 and 3-6, Parent 1 completed the Hendrick relationship quality scale: How well does your partner meet your needs?  How good is your relationship compared to most?  How often do you wish you hadn't married or lived together?  To what extent has your marriage or relationship met your original expectations?  How much do you love your partner?  How many problems are there in your relationship? | | Responses ranged from 1-most negative to 5-most positive. Items 3 and 6 were reverse coded and the mean of all items was computed at each timepoint. | 5 variables; one at each timepoint. Continuous. |  |
|  | | Parent stressful life events | | At Waves 1-7, Parent 1 was asked whether they, or their partner, had experienced a range of stressful life events in the last year:  You suffered a serious illness, injury or assault  A serious illness or assault happened to a close relative  Your parent, partner or child died  A close family friend or another relative (aunt, cousin, grandparent) died  You broke off a steady romantic relationship  You had a serious problem with a close friend, neighbour or relative  You had a crisis or serious disappointment in your work career  You thought you would soon lose your job  You lost your job, but not from choice (sacked/redundant, contract ended)  You lost your job, but not from choice (sacked/redundant, contract ended)  You were seeking work unsuccessfully for more than one month  You were seeking work unsuccessfully for more than one month  You had a major financial crisis  You had problems with the police and a court appearance  Something you valued was lost or stolen  Someone in your household had an alcohol or drug problem  Someone in your household had an alcohol problem  Someone in your household had a drug-use problem  Your partner lost his/her job, but not from choice (sacked/redundant, contract ended)  Your partner was seeking work unsuccessfully for more than one month  You had a separation due to relationship or marital difficulties | | A total score of the number of yes responses at each timepoint was computed. | 8 variables; one at each timepoint, and one summary variable reflecting total number of parent stressful life events across waves. Continuous. |  |
|  | | Difficulty of life at present | | At Waves 1-7, Parent 1 was asked: “How difficult do you feel your life is at present?” | | Response options were 1 No problems or stress; 2 Few problems or stresses; 3 Some problems and stresses; 4 Many problems and stresses; 5 Very many problems and stresses | 7 variables: one at each timepoint. Ordinal. |  |
|  | | Social support available to parent | | At Waves 2, 3, and 5, parent 1 was asked: “How often do you feel you need support but can't get it from anyone?” | | 1 Very often; 2 Often; 3 Sometimes; 4 Never. | 3 variables; one at each timepoint. Ordinal. |  |
|  | | Couple arguments | | At Waves 1-7, Parent 1 was asked: “How often...Do you and your partner disagree about basic child-rearing issues  How often...Is your conversation with your partner awkward or stressful  How often...Do you and your partner argue  How often...Is there anger or hostility between you and your partner” | | Response options: 1 Never; 2 Rarely; 3 Sometimes; 4 Often; 5 Always. The mean across all items at each timepoint was created, with higher scores reflecting greater frequency of arguments. | 7 variables: one at each timepoint. Continuous. |  |
| **Child mental health / behaviours** | | | | | |  |  |  |
|  | | Early onset of alcohol use | | At Wave 6 (age 14-15), child was asked whether they had ever had even part of an alcoholic drink. | | Response options were: 1 No; 2 Yes, just a few sips; 3 Yes, I have had fewer than 10 alcoholic drinks in my life; 4 Yes, I have had 10 or more alcoholic drinks in my life. Early onset of alcohol use was coded as those who endorsed response options 3 or 4 i.e. those who reported lifetime more than just a few sips of alcohol. | 1 variable. Binary yes/no. |  |
|  | | Frequent adolescent alcohol use | | Participants were asked about their alcohol consumption at Wave 7 (age 16/17), including the number of alcoholic drinks consumed in the last seven days. | | Those who consumed at least one alcoholic drink in the last seven days were coded as engaging in past week alcohol use. Those who did not drink in the past seven days, past month, past year, or never, were coded as no. | 1 variable. Binary yes/no. |  |
|  | | Lifetime cannabis use | | At Wave 7 (age 16/17), participants reported whether they had ever used cannabis. | | Binary yes/no. | 1 variable. Binary yes/no. |  |
|  | | Lifetime drug use | | At Wave 7 (age 16/17), participants reported whether they had ever used other drugs (and given the examples speed, ecstasy, LSD, ice, cocaine, heroin. | | Binary yes/no. | 1 variable. Binary yes/no. |  |
|  | | Attention deficit hyperactivity disorder (ADHD) | | From Wave 1-7 (ages 4/5 – 16/17), Parent 1 responded whether their child had any of these ongoing problems, with Attention Deficit Disorder (ADD or ADHD) listed as one possible condition. | | A composite variable was created such that children with any timepoint where ADHD was reported was coded as yes to lifetime ADHD. | 1 variable. Binary yes/no. |  |
|  | | Conduct problems | | Measured using the conduct problems subscale of the child self-report Strengths and Difficulties Questionnaire (SDQ). At Waves 4-7 (ages 10/11 – 16/17) the child rated the frequency of conduct problems (e.g., Often fights with other children or bullies them). | | Three-point scale: 1 = Not true, 2 = somewhat true, 3 = certainly true. Scores were rescaled from 0-2, and a total score was created with a range from 0-10. | 4 variables; one at each timepoint. Continuous. |  |
|  | | Hyperactivity | | Measured using the hyperactivity subscale of the child self-report Strengths and Difficulties Questionnaire (SDQ). At Waves 4-7 (ages 10/11 – 16/17) the child rated the frequency of symptoms of hyperactivity (e.g., difficulty concentrating, easily distracted). | | Three-point scale: 1 = Not true, 2 = somewhat true, 3 = certainly true. Scores were rescaled from 0-2, and a total score was created with a range from 0-10. | 4 variables; one at each timepoint. Continuous. |  |
|  | |  | Delinquency | | Measure adapted from the Self-Report Delinquency Scale [4]. At Waves 5-7, child rated how often they had engaged in 17 various negative social behaviours, such as skipping school, purposely damaging others’ property, theft etc. in the past 12 months. | Five-point scale: 0 Not at all; 1 Once; 2 Twice; 3 Three times; 4 Four times; 5 Five or more times.  Total score at each wave was created for participants with fewer than 3 missing items on the 17-item scale. | 3 variables; one at each timepoint. Continuous. | |
|  | |  | Emotional problems | | Measured using the emotional problems subscale of the child self-report Strengths and Difficulties Questionnaire (SDQ). At Waves 4-7 (ages 10/11 – 16/17) the child rated the frequency of emotional problems (e.g., often unhappy, depressed, or tearful, worry a lot, nervous in new situations). | Three-point scale: 1 = Not true, 2 = somewhat true, 3 = certainly true. Scores were rescaled from 0-2, and a total score was created with a range from 0-10. | 4 variables; one at each timepoint. Continuous. | |
|  | Neuroticism | | | At Wave 7 (age 16/17), measured with the Big Five Inventory-10. Two items measured neuroticism: I see myself as someone who is relaxed, handles stress well (reversed coded) and I see myself as someone who gets nervous easily. | | Response options ranged from 1 Disagree strongly; 2 Disagree a little; 3 Neither agree nor disagree; 4 Agree a little; 5 Agree strongly. Mean score across items was calculated. | 1 variable. Continuous. |  |
|  | Extraversion | | | At Wave 7 (age 16/17), measured with the Big Five Inventory-10. Two items measured extraversion: I see myself as someone who is reserved; keeps thoughts and feelings to self (reversed), and I see myself as someone who is outgoing, sociable. | | Response options ranged from 1 Disagree strongly; 2 Disagree a little; 3 Neither agree nor disagree; 4 Agree a little; 5 Agree strongly. Mean score across items was calculated. | 1 variable. Continuous. |  |
|  | Openness | | | At Wave 7 (age 16/17), measured with the Big Five Inventory-10. Two items measured openness: I see myself as someone who doesn’t like artistic things (plays, music). I see myself as someone who has an active imagination | | Response options ranged from 1 Disagree strongly; 2 Disagree a little; 3 Neither agree nor disagree; 4 Agree a little; 5 Agree strongly. Mean score across items was calculated. | 1 variable. Continuous. |  |
|  | Agreeableness | | | At Wave 7 (age 16/17), measured with the Big Five Inventory-10. How well do the following statements describe your personality? I see myself as someone who is generally trusting. I see myself as someone who tends to find fault with others (reversed) | | Response options ranged from 1 Disagree strongly; 2 Disagree a little; 3 Neither agree nor disagree; 4 Agree a little; 5 Agree strongly. Mean score across items was calculated. | 1 variable. Continuous. |  |
|  | Conscientiousness | | | At Wave 7 (age 16/17), measured with the Big Five Inventory-10. How well do the following statements describe your personality? I see myself as someone who tends to be lazy (reversed). I see myself as someone who does things carefully and completely. | | Response options ranged from 1 Disagree strongly; 2 Disagree a little; 3 Neither agree nor disagree; 4 Agree a little; 5 Agree strongly. Mean score across items was calculated. | 1 variable. Continuous. |  |
|  | Self-harm ideation | | | At Waves 6-7, the study child was asked: During the past 12 months have you thought about hurting yourself on purpose in any way? | | Binary yes/no. | 2 variables: one at each timepoint. Binary yes/no. |  |
|  | Self-harm | | | At Waves 6-7, the study child was asked: During the past 12 months have you hurt yourself on purpose in any way? | | Binary yes/no. | 2 variables: one at each timepoint. Binary yes/no. |  |
|  | Suicidal ideation | | | At Waves 6-7, the study child was asked: During the past 12 months did you ever seriously consider attempting suicide? | | Binary yes/no. | 2 variables: one at each timepoint. Binary yes/no. |  |
|  | Suicide plan | | | At Waves 6-7, the study child was asked: During the past 12 months did you make a plan about how you would attempt suicide? | | Binary yes/no. | 2 variables: one at each timepoint. Binary yes/no. |  |
|  | Suicide attempt | | | At Waves 6-7, the study child was asked about the number of times attempted suicide in past 12 months. | | 0 0 times; 1 1 time; 2 2 or 3 times; 3 4 or 5 times; 4 6 or more times. Anything more than 0 was coded as a suicide attempt. | 2 variables: one at each timepoint. Binary yes/no. |  |
|  | Religiosity | | | At Wave 6, the study child was asked: “Are you active in a religious or spiritual group, such as regularly going to services, Sunday school or a religious youth club?” | | Options were Yes or No. | 1 variable. Binary yes/no. |  |
|  | Sleep problems | | | Waves 1-6, parent 1 reported whether the child had any of the following problems on 4 or more nights a week, that is, more than half the time?  Wheezing or asthma  Snoring or difficulty breathing  Difficulty getting off to sleep at night  Not happy to sleep alone  Waking during the night  Bed wetting  Nightmares or night terrors  Seeming tired in the morning  Other problems | | Binary yes/no to any sleep problems. | 6 variables: one at each timepoint. Binary yes/no. |  |
|  | Sleep quantity | | | At Waves 4-7, the study child was asked: “During the last month, do you think you usually got enough sleep?” | | 1 Plenty; 2 Just enough; 3 Not quite enough; 4 Not nearly enough. | 4 variables; one at each timepoint. Ordinal. |  |
|  | Anxiety | | | At Waves 5-7, the study child completed the Spence Anxiety Scale.  Please select the word that shows how often each of these things happen to you. There are no right or wrong answers. I worry about things  I feel afraid  I feel afraid that I will make a fool of myself in front of people  I worry that something bad will happen to me  I feel nervous. | | 1 Never; 2 Sometimes; 3 Often; 4 Always. A total score was computed at each timepoint, with higher scores indicating greater levels of anxiety. | 3 variables; one at each timepoint. Continuous. |  |
|  | Depression | | | At Waves 5-7, the study child completed the Short mood and feelings questionnaire. For each question please select how much you have felt or acted this way in the past two weeks.  I felt miserable or unhappy.  I didn't enjoy anything at all  I felt so tired I just sat around and did nothing  I was very restless  I felt I was no good anymore  I cried a lot  I found it hard to think properly or concentrate  I hated myself  I was a bad person  I felt lonely  I thought nobody really loved me  I thought I could never be as good as other kids  I did everything wrong. | | 1 True; 2 Sometimes; 3 Not true. All items were reverse coded and then summed to create a total score, with higher scores reflecting greater depressive symptoms. | 3 variables: one at each timepoint. Continuous. |  |
|  | Overall happiness | | | At Waves 5-7, the child was asked “In general, I am happy with how things are for me in my life right now.” | | Response options were: 1 Strongly disagree; 2 Disagree; 3 Neither agree nor disagree; 4 Agree; 5 Strongly agree | 3 variables; one at each timepoint. Ordinal. |  |
|  | Social support | | | At Waves 4-5, the child was asked If you had a problem, who would you talk to about it? | | Those who responded “no one” were coded as having no social support. | 2 variables, one at each timepoint. Binary. |  |
| **School** | | | |  | |  |  |  |
|  | | Overall school achievement | | Measured at Waves 2-6 (ages 6-14) from an LSAC developed scale. Concept derived from the National Longitudinal Survey of Children and Youth (NLSCY): Cycle 4 (Survey Instruments, 2000-2001, Teacher Questionnaire). | | The parent described the child’s overall achievement at school, from 1-Excellent to 5-Well below average. Scores were reversed such that higher scores reflected greater school achievement. | 1 variable. From ages 10-14, computed a maximum score for each participant which was their highest rating of school achievement. |  |
|  | | School belonging | | Measured using the Psychological Sense of School Membership Scale (PSSM), collected at waves 5-7 (ages 12/13 – 16/17). 12-item scale that assesses a child’s sense of belonging and acceptance at school e.g. I can really be myself at this school. | | Response options were from 1 Not at all true;2 Not very true;3 Neither not at all true nor completely true; 4 Somewhat true;5 Completely true. Four items were reverse coded and a total score was calculated whereby higher scores reflected greater school belonging. | 4 variables. Total score at each timepoint. Continuous. |  |
|  | | Academic pressure from parents | | At wave 7 (age 16/17), the study child was asked, “In the past 2 years, how often did you feel that your parents expected too much from you with regards to your performance in your studies?” | | Response options ranged from 1 All of the time; 2 Most of the time; 3 Some of the time; 4 Rarely; 5 Never. Those who endorsed options 1 or 2 were coded as high academic pressure from parents. | 1 variable. Binary yes/no. |  |
|  | | Number of different schools attended | | At Wave 6, Parent 1 was asked: “How many different schools has study child ever attended, since beginning full-time schooling?” | | Number. | 1 variable. Continuous. |  |
|  | | Parent interest in education | | At Waves 5-6, the child was asked “How much interest does your mother show towards your learning and education?” And the same question for the father. | | 1 A lot of interest; 2 Some interest; 3 Not much interest; 4 No interest at all. Variables relating to the mother were recoded such that 3 and 4 were collapsed into a single category (Not interested) to reduce sparsity for the knn imputation procedure. | 4 variables, one at each timepoint for mother and one at each timepoint for father. Ordinal. |  |
| **Peers** | | | |  | |  |  |  |
|  | | Peer deviancy | | Measured at Waves 5-7 with 7 items sourced from the ‘What my Friends are Like Questionnaire’ and the ATP. These asked how many of the child’s friends engaged in negative social behaviours e.g. they are mean to other kids, they get in trouble at school. | | Scores were summed to create a total score, with higher scores reflecting a lower number of deviant peers. | 3 variables; one at each timepoint. Continuous score. |  |
|  | | Peer problems | | Measured with child self-report from Wave 4-7 using the SDQ peer problems scale, which asked participants to think over the last 6 months and respond to items such as “Other people my own age generally like me.” | | 1 Not true; 2 Somewhat true; 3 Certainly true. Scores were rescaled from 0-2, and a total score was created with a range from 0-10. Two items were reverse coded, such that higher scores indicated greater peer problems. | 5 variables. Continuous score at each timepoint. |  |
|  | | Discrimination | | At waves 5-7, the study child reported whether they had been treated badly or unfairly in the last 6 months due to a range of attributes, e.g. skin colour, accent, sexual identity, disability. 5 items were asked at age 12, 8 items at age 14, 9 items at age 16. | | Total number of discrimination events at each timepoint, plus a summary variable reflecting the total number of events across all timepoints. | 4 variables. One at each timepoint, plus a summary variable across all timepoints. Continuous. |  |
|  | | Bullying victimisation | | At Waves 6-7, study child was asked about their experiences of bullying victimisation in the last 12 months. Example items included someone hit or kicked me on purpose, threatened to hurt me, said mean things to me or called me names, tried to keep others from being my friend, did not let me join in what they were doing, hurt me or tried to hurt me with a weapon, stole my things to be mean to me, forced me to do something I didn’t want to do. 11 items at Wave 6, 9 items at wave 7. | | Yes/no to each experience. The number of experiences were summed to create a total score at each timepoint. | 2 variables, one at each timepoint. Continuous. |  |
|  | | Bullying perpetration | | At Waves 6-7, study child was asked about whether they had perpetrated the acts above to others. 11 items at Wave 6, 9 items at wave 7. | | Yes/no to each experience. The number of experiences were summed to create a total score at each timepoint. | 2 variables, one at each timepoint. Continuous. |  |
|  | | Peer communication | | At Waves 5-7, the child completed the Inventory of Peer and Parent Attachment communication subscale. Items include: My friends sense when I'm upset about something, My friends encourage me to talk about my difficulties, I tell my friends about my problems and troubles, If my friends know something is bothering me, they ask me about it | | 1 Almost always true;2 Often true; 3 Sometimes true;4 Seldom true;5 Almost never true. Scores were summed to create a total score, with higher scores reflecting poorer peer communication. | 3 variables; one at each timepoint. Continuous. |  |
|  | | Peer trust | | At Waves 5-7, the child completed the Inventory of Peer and Parent Attachment communication subscale. Items include: My friends listen to what I say, I feel my friends are good friends, I trust my friends, My friends respect my feelings. | | 1 Almost always true;2 Often true; 3 Sometimes true;4 Seldom true;5 Almost never true. Scores were summed to create a total score, with higher scores reflecting poorer peer trust. | 3 variables; one at each timepoint. Continuous. |  |

Supplementary methods: Missing data and Imputation

For the K-cohort used in the current study, factors associated with greater odds of non-response across survey follow-up waves include lower birth weight, not being the first-born child, higher conduct problems, academic problems, neither parent having a bachelor degree or above, both parents with a medical condition or disability, parent smoking status, parental consistency, parent argumentativeness, parent stressful life events, greater number of siblings in the household, households where the main source of income was not government benefits, moving house in the previous two years, not being interviewed by the same interviewer in a previous wave. Further information can be found in Technical Paper 23 [5].

Missing data was handled through k-nearest neighbour (KNN) imputation using the VIM package in R. KNN imputation is a non-parametric method used to handle missing data by estimating the missing values based on the similarity of observations. Specifically, for each missing value in a dataset, KNN identifies the k most similar observations, or "neighbours," based on a chosen distance metric (typically Euclidean distance for continuous variables or Hamming distance for categorical variables). The missing value is then imputed using a weighted average or majority vote from these nearest neighbours, depending on whether the variable is continuous or categorical. KNN imputation is advantageous as it leverages the relationships between variables to provide context-specific estimates, preserving patterns in the data and potentially reducing bias introduced by missing values. Prior to imputing, all continuous variables were scaled using z score standardisation, as distance-based methods like KNN are sensitive to differences in scale and magnitude between variables. Scaling prevents variables with larger ranges from disproportionately influencing the distance calculations. Binary variables were converted to numeric variables for imputation. The value of k was set at 5 to optimize imputation accuracy while balancing the trade-off between local precision and global smoothing. All presented analyses pertain to the imputed data, except sample characteristics as shown in Table 2.

Supplementary methods: Hyperparameter tuning

Hyperparameter tuning was conducted within a nested cross-validation framework to optimize three base learners integrated into the SuperLearner ensemble for predicting risky alcohol consumption. For the random forest algorithm (SL.ranger), tuning parameters included the number of variables randomly selected at each split (mtry), set to the square root and one-third of the total number of predictors (221), and the minimum node size (min.node.size), tested at 5 and 10, with the number of trees fixed at 200 and a maximum depth of 10. For the elastic net regularized regression (SL.glmnet), the mixing parameter (alpha) was tuned across values from 0 (Ridge regression) to 1 (LASSO) in increments of 0.2, paired with a penalty parameter (lambda) ranging from 0.01 to 0.05 over 10 evenly spaced values. For the support vector machine with a radial basis function kernel (SL.ksvm), the regularization parameter (C) was evaluated at 0.1, 1, and 10, and the kernel width (sigma) at 0.01, 0.1, and 1. Hyperparameter combinations were assessed using 10-fold cross-validation within each outer training fold, optimizing the area under the receiver operating characteristic curve (ROC-AUC) as the performance metric. Optimal parameters were selected for each learner per fold and applied in custom SuperLearner wrappers, which were combined using non-negative least squares weighting to form the final ensemble predictions.

Supplementary Table 3: SuperLearner weights and AUC for the ensemble for all 15 folds, as well as the average weighting across all 15 folds.

| Fold | Ranger | Glmnet | KSVM | Ensemble AUC |
| --- | --- | --- | --- | --- |
| 1 | 0.1595356 | 0.3650152 | 0.4754492 | 0.7888 |
| 2 | 0.05605807 | 0.503463 | 0.4404789 | 0.8116 |
| 3 | 0.20204454 | 0.4101544 | 0.3878011 | 0.7918 |
| 4 | 0.00915339 | 0.547687 | 0.4431596 | 0.7680 |
| 5 | 0.16215914 | 0.597215 | 0.2406259 | 0.7856 |
| 6 | 0.23183587 | 0.3135427 | 0.4546215 | 0.8204 |
| 7 | 0.05403494 | 0.466516 | 0.479449 | 0.8034 |
| 8 | 0.06561307 | 0.5731196 | 0.3612673 | 0.7924 |
| 9 | 0.1131784 | 0.52526 | 0.3615616 | 0.7750 |
| 10 | 0.08211943 | 0.5027356 | 0.415145 | 0.7882 |
| 11 | 0.1234188 | 0.4357717 | 0.4408095 | 0.7736 |
| 12 | 0.22976447 | 0.3518842 | 0.4183513 | 0.8059 |
| 13 | 0.29078129 | 0.3851923 | 0.3240264 | 0.7925 |
| 14 | 0.21672405 | 0.3556778 | 0.4275981 | 0.8047 |
| 15 | 0.09931068 | 0.5815291 | 0.3191603 | 0.7821 |
| Mean across all folds | 0.139715 | 0.460984 | 0.399300 | 0.7922 |

Supplementary Table 4. Feature importance, normalised and weighted by SuperLearner coefficients and aggregated across folds for all 220 predictors.

| **Feature** | **Weighted Mean** | **Weighted SD** |
| --- | --- | --- |
| Weekly drinking (age 16) | 0.99925097 | 0.002901 |
| Lifetime Cannabis Use | 0.44625379 | 0.05452562 |
| Financial stress | 0.4203031 | 0.03705029 |
| Female | 0.36512711 | 0.08061375 |
| Male | 0.3442192 | 0.05093263 |
| ADHD | 0.24809721 | 0.05555495 |
| Pre-natal alcohol exposure | 0.24807747 | 0.04023639 |
| Housing insecurity | 0.24328065 | 0.05270023 |
| Religious involvement | 0.23832599 | 0.04286568 |
| Parent 1 alcohol use problem | 0.21496378 | 0.04816134 |
| Conduct problems (16) | 0.19304537 | 0.04394482 |
| Bully victimisation (16) | 0.1927689 | 0.04903178 |
| Anxiety (16) | 0.18138694 | 0.05219186 |
| Moral peers (16) | 0.1799466 | 0.03000665 |
| Neuroticism (16) | 0.17742442 | 0.04458989 |
| Family SES (4) | 0.174376 | 0.0355995 |
| Lifetime other drug use | 0.16979163 | 0.05638454 |
| Parental monitoring (14) | 0.16660787 | 0.04208602 |
| Parent 1 self-efficacy (12) | 0.16515154 | 0.04821889 |
| Moral peers (14) | 0.16048207 | 0.04854947 |
| Highest household qualification - university | 0.15962346 | 0.04924393 |
| Sleep problems (4) | 0.15427091 | 0.03812342 |
| Anxiety (12) | 0.15190427 | 0.04537 |
| Sleep quantity (12) | 0.14956466 | 0.03898145 |
| Extraversion (16) | 0.14645907 | 0.02829698 |
| Parent 1 mental health disorder | 0.1375079 | 0.04118046 |
| Area-level socioeconomic advantage and disadvantage (14) | 0.13585229 | 0.03731103 |
| Suicidal ideation (16) | 0.13319076 | 0.034867 |
| Anxiety (14) | 0.13239973 | 0.04039778 |
| Peer problems (14) | 0.13131958 | 0.03374413 |
| Hyperactivity (14) | 0.1249177 | 0.02761883 |
| Consistent parenting (14) | 0.12116032 | 0.03510853 |
| Emotional problems (12) | 0.12073171 | 0.04479356 |
| Delinquency (16) | 0.11989434 | 0.03052048 |
| Self-harm (14) | 0.11968909 | 0.04971845 |
| Unsupervised time (14) | 0.11434959 | 0.03649017 |
| Angry parenting (8) | 0.11307024 | 0.03056943 |
| Parent support to child (14) | 0.11184074 | 0.0300591 |
| Parent 1 psychological distress (14) | 0.11116928 | 0.03417558 |
| Parent couple arguments (12) | 0.11073607 | 0.0298309 |
| Delinquency (14) | 0.1093479 | 0.02621065 |
| Parent 1 self-efficacy (14) | 0.10784511 | 0.03512424 |
| Parent relationship quality (4) | 0.10712363 | 0.03764334 |
| Unsupervised time (12) | 0.10697492 | 0.04430654 |
| Emotional problems (16) | 0.10696974 | 0.04505476 |
| Bully perpetration (16) | 0.10593309 | 0.03293864 |
| Hyperactivity (16) | 0.10505133 | 0.04361638 |
| Parent 1 stressful life events | 0.10462779 | 0.03007919 |
| School belonging (14) | 0.10401113 | 0.02466315 |
| Parent 1 highest qualification | 0.10303644 | 0.02834618 |
| Parent 1 stressful life events (16) | 0.10234423 | 0.02724831 |
| Hyperactivity (12) | 0.10229051 | 0.03269716 |
| Parent couple arguments (6) | 0.10220263 | 0.04018675 |
| Parent 1 stressful life events (14) | 0.10173014 | 0.03392886 |
| Openness (16) | 0.1015262 | 0.0296791 |
| Parental warmth (10) | 0.10150551 | 0.02586396 |
| Parent support to child (16) | 0.10046784 | 0.02768987 |
| Peer problems (10) | 0.10007151 | 0.02409101 |
| Peer trust (14) | 0.0999285 | 0.03328096 |
| Parental warmth (6) | 0.0993625 | 0.03409287 |
| Conscientiousness (16) | 0.09883718 | 0.02796248 |
| Peer communication (12) | 0.09857576 | 0.02251004 |
| Peer trust (12) | 0.09833968 | 0.02869548 |
| Highest household education | 0.09744952 | 0.03562823 |
| Conduct problems (14) | 0.09742132 | 0.02708689 |
| Parent 1 stressful life events (12) | 0.09737752 | 0.02545209 |
| Angry parenting (14) | 0.09668701 | 0.02896505 |
| Sleep problems (12) | 0.0966562 | 0.03437309 |
| Out of home activities (6) | 0.09647144 | 0.03303102 |
| Discrimination (16) | 0.09606296 | 0.02629005 |
| Parent 1 difficulty of life (16) | 0.0957848 | 0.03360164 |
| Number of schools attended | 0.09569924 | 0.03553391 |
| Consistent parenting (12) | 0.09487863 | 0.02342166 |
| Close to mum (12) | 0.09446274 | 0.03269878 |
| Close to mum (16) | 0.09373452 | 0.02624013 |
| Peer problems (16) | 0.09350104 | 0.0333181 |
| Peer communication (16) | 0.09335217 | 0.02406259 |
| Overall happiness (16) | 0.0932325 | 0.03215133 |
| Home activities (4) | 0.09299557 | 0.02118985 |
| Parent 1 difficulty of life (12) | 0.09283973 | 0.02788803 |
| Suicidal ideation (14) | 0.09258544 | 0.04314509 |
| Suicide attempt (14) | 0.09252965 | 0.02701052 |
| Lack of social support (10) | 0.09183649 | 0.03169526 |
| Parent 1 difficulty of life (6) | 0.09127024 | 0.02295979 |
| Financial stress (14) | 0.0911519 | 0.02850177 |
| Overall happiness (12) | 0.09090209 | 0.03117752 |
| Financial stress (12) | 0.09067358 | 0.03075523 |
| Parent 1 psychological distress (16) | 0.0905396 | 0.03700855 |
| Sleep quantity (16) | 0.08949193 | 0.02864755 |
| Consistent parenting (4) | 0.08838581 | 0.03006161 |
| Discrimination (14) | 0.0882766 | 0.01805285 |
| Bully perpetration (14) | 0.08789791 | 0.03498363 |
| Angry parenting (12) | 0.08789129 | 0.01837822 |
| Housing insecurity (16) | 0.08785373 | 0.04381512 |
| Dad interest in child’s education (14) | 0.08784127 | 0.02774102 |
| Peer communication (14) | 0.08753328 | 0.03114295 |
| Suicide attempt (16) | 0.08751167 | 0.02812449 |
| Peer problems (12) | 0.08746807 | 0.03068699 |
| Area-level socioeconomic advantage and disadvantage (12) | 0.08668256 | 0.02324357 |
| Mum interest in child’s education (12) | 0.08662267 | 0.02979992 |
| Self-harm ideation (14) | 0.0863621 | 0.02698566 |
| Parental monitoring (12) | 0.08618317 | 0.02376552 |
| Parent 1 stressful life events (4) | 0.08606093 | 0.02759407 |
| Bully victimisation (14) | 0.08603876 | 0.0255592 |
| Parent couple arguments (8) | 0.08599346 | 0.03258574 |
| Moral peers (12) | 0.08580775 | 0.02174694 |
| Out of home activities (8) | 0.08567074 | 0.02598391 |
| Depressive symptoms (16) | 0.08561035 | 0.03183987 |
| Lack of social support (12) | 0.08537516 | 0.02736242 |
| Home activities (10) | 0.08533682 | 0.03172435 |
| Parent 1 Psychological Distress (10) | 0.08529071 | 0.02511654 |
| Parent 1 Psychological Distress (4) | 0.08496819 | 0.02662475 |
| Parent 1 difficulty of life (14) | 0.08491093 | 0.03177583 |
| Mum interest in child’s education (14) | 0.0847554 | 0.02819858 |
| Conduct problems (10) | 0.08462684 | 0.01926468 |
| Angry parenting (4) | 0.08436863 | 0.03664199 |
| Depressive symptoms (12) | 0.08416143 | 0.03268846 |
| Out of home activities (4) | 0.08415482 | 0.02185052 |
| Self-harm ideation (16) | 0.08413125 | 0.0288418 |
| Neighbourhood liveability (4) | 0.08353861 | 0.04121146 |
| Sleep problems (10) | 0.08343364 | 0.02374501 |
| Overall happiness (14) | 0.08332496 | 0.04307395 |
| Parent 2 highest qualification | 0.08279564 | 0.03185754 |
| Domestic violence (12) | 0.0826209 | 0.02997544 |
| Angry parenting (10) | 0.08246393 | 0.02804777 |
| Parent 1 drug use disorder | 0.08192742 | 0.03493068 |
| Highest household qualification - diploma | 0.08139708 | 0.03259411 |
| Consistent parenting (6) | 0.08110243 | 0.02939358 |
| Elevated emotional problems (10) | 0.08097591 | 0.02594045 |
| Parent relationship quality (12) | 0.08080945 | 0.02492073 |
| Parent support to child | 0.08066751 | 0.03209112 |
| Domestic violence (4) | 0.0803315 | 0.02248194 |
| Parent couple arguments (10) | 0.08030023 | 0.02849891 |
| Consistent parenting (8) | 0.07976167 | 0.03553514 |
| School belonging (16) | 0.07975133 | 0.02784519 |
| Dad interest in child’s education (12) | 0.07936304 | 0.02868887 |
| Self-harm (16) | 0.07906158 | 0.0274077 |
| Transgender | 0.07887656 | 0.02719033 |
| Parent relationship quality (8) | 0.07870818 | 0.02045717 |
| Sleep quantity (10) | 0.07843367 | 0.02360567 |
| Parent couple arguments (4) | 0.0783452 | 0.02852934 |
| Parental warmth | 0.07829753 | 0.02384104 |
| Parent 1 self-efficacy (4) | 0.0778196 | 0.023365 |
| Parent 1 self-efficacy (16) | 0.07774629 | 0.0288164 |
| Delinquency (12) | 0.07771932 | 0.02734481 |
| Neighbourhood liveability (14) | 0.07770908 | 0.01860559 |
| Housing insecurity (14) | 0.0776701 | 0.02300458 |
| Academic pressure from parents | 0.07759417 | 0.02689711 |
| Financial stress (16) | 0.07718199 | 0.02627344 |
| Parent 1 financial stress (10) | 0.07711298 | 0.02664447 |
| Domestic violence (8) | 0.07702409 | 0.02326356 |
| Parent 1 difficulty of life (8) | 0.07682923 | 0.02691559 |
| Suicide plan (14) | 0.07676007 | 0.02294617 |
| Parent 1 difficulty of life (4) | 0.07675446 | 0.02587263 |
| Close to mum (14) | 0.07629463 | 0.0266994 |
| Housing insecurity (12) | 0.07629432 | 0.03170767 |
| Parent 1 self-efficacy (10) | 0.07619453 | 0.02103783 |
| School belonging (12) | 0.0761609 | 0.01895992 |
| Parental monitoring (8) | 0.07608173 | 0.03106075 |
| Parental warmth (4) | 0.07592671 | 0.02216194 |
| Out of home activities (12) | 0.07557027 | 0.02259676 |
| Parent couple arguments (14) | 0.07526202 | 0.04160365 |
| Consistent parenting (10) | 0.07481376 | 0.0205022 |
| Parent relationship quality (10) | 0.07476353 | 0.02981732 |
| Support available to parent (4) | 0.07474077 | 0.0286063 |
| Support available to parent (6) | 0.07462997 | 0.01915875 |
| Discrimination (12) | 0.07461607 | 0.02382159 |
| Parent 1 stressful life events (10) | 0.07455827 | 0.03117677 |
| Home activities (8) | 0.07426872 | 0.03605321 |
| Neighbourhood liveability (6) | 0.07318769 | 0.02058675 |
| Parent 1 Psychological Distress (8) | 0.07313848 | 0.02232024 |
| Domestic violence (14) | 0.07313303 | 0.02107617 |
| Area-level socioeconomic advantage and disadvantage (14) | 0.07304618 | 0.02046691 |
| Peer trust (16) | 0.07287527 | 0.023619 |
| Angry parenting (16) | 0.07284273 | 0.03195391 |
| Early onset alcohol use | 0.07223327 | 0.02049687 |
| Discimination | 0.07206919 | 0.0185949 |
| Neighbourhood liveability (10) | 0.07194511 | 0.01826829 |
| Consistent parenting (16) | 0.07182938 | 0.02654971 |
| Parent 1 Psychological Distress (6) | 0.07181601 | 0.02496733 |
| Depressive symptoms (14) | 0.07170983 | 0.0301357 |
| Support available to parent (12) | 0.07157722 | 0.02854093 |
| Number of homes lived in | 0.07157322 | 0.03105899 |
| Close to dad (12) | 0.07155841 | 0.02484171 |
| Parental warmth (14) | 0.07150519 | 0.03316533 |
| Parent 1 stressful life events (8) | 0.07130654 | 0.02533778 |
| Angry parenting (6) | 0.07127443 | 0.02887725 |
| Sleep quantity (14) | 0.07109965 | 0.02319666 |
| Area-level socioeconomic advantage and disadvantage (10) | 0.07069341 | 0.02320696 |
| Parent 1 Psychological Distress (12) | 0.07055423 | 0.02808935 |
| Domestic violence | 0.06991196 | 0.02310178 |
| Area-level socioeconomic advantage and disadvantage (4) | 0.06982735 | 0.02592089 |
| Close to dad (14) | 0.06962237 | 0.02889479 |
| Support available to parent (8) | 0.06939417 | 0.02305681 |
| Parental monitoring (10) | 0.06928936 | 0.02738433 |
| Parent separation (16) | 0.06893488 | 0.02612362 |
| School achievement | 0.0688451 | 0.03382752 |
| Home activities (6) | 0.06856439 | 0.03214989 |
| Parent 1 difficulty of life (10) | 0.06854754 | 0.02052211 |
| Area-level socioeconomic advantage and disadvantage (6) | 0.06848691 | 0.02296779 |
| Conduct problems (12) | 0.06829739 | 0.0185043 |
| Sleep problems (8) | 0.06821301 | 0.01978814 |
| Parental warmth (12) | 0.06795276 | 0.02375172 |
| Parental warmth (8) | 0.0674595 | 0.01887054 |
| Parent 1 self-efficacy (6) | 0.06734057 | 0.01906395 |
| Sleep problems (14) | 0.06694133 | 0.02713731 |
| Agreeableness (16) | 0.06679805 | 0.02625175 |
| Parent 1 self-efficacy (8) | 0.06677266 | 0.01771047 |
| Sleep problems (6) | 0.06571643 | 0.01859423 |
| Emotional problems (14) | 0.06542383 | 0.02530617 |
| Area-level socioeconomic advantage and disadvantage (8) | 0.06481838 | 0.02145811 |
| Out of home activities (10) | 0.06478222 | 0.0265126 |
| Domestic violence (6) | 0.06474537 | 0.02101976 |
| Parent 1 stressful life events (6) | 0.06418911 | 0.02838138 |
| Domestic violence (10) | 0.06380824 | 0.02017575 |
| Parent relationship quality (14) | 0.06363991 | 0.02509729 |
| Suicide plan (16) | 0.06342173 | 0.02453849 |
| Out of home activities (14) | 0.06337754 | 0.02889543 |
| Hyperactivity (10) | 0.06333402 | 0.02409268 |
| Neighbourhood liveability (8) | 0.06153919 | 0.02037779 |

**Results – Sensitivity analysis**

Results of the sensitivity analysis removing predictors capturing previous substance use in adolescence (weekly drinking at age 16, cannabis use, and other drug use) are reported below. Averaging across folds, ksvm emerged as the most effective individual algorithm (coefficient 0.466), followed by glmnet (coefficient 0.342), and random forest (coefficient 0.192). Weights per fold are presented in Supplementary Table 5. Model performance for SuperLearner yielded a mean AUC of 0.771 across the 15 folds, which reflects moderate accuracy, slightly reduced compared to the AUC in the main analysis of 0.7922. While SuperLearner outperformed individual algorithms, the difference was small (Supplementary Table 6).

Supplementary Table 5: SuperLearner weights for all 15 folds, as well as the average weighting across all 15 folds.

| Fold | Ranger | Glmnet | KSVM |
| --- | --- | --- | --- |
| 1 | 0.1855761 | 0.2672557 | 0.5471682 |
| 2 | 0.17746724 | 0.3468218 | 0.475711 |
| 3 | 0.26532106 | 0.2722038 | 0.4624751 |
| 4 | 0.11358681 | 0.3193109 | 0.5671022 |
| 5 | 0.08574873 | 0.516613 | 0.3976382 |
| 6 | 0.2499519 | 0.308542 | 0.4415061 |
| 7 | 0.21137167 | 0.2258799 | 0.5627484 |
| 8 | 0.09322441 | 0.4973277 | 0.4094479 |
| 9 | 0.25207696 | 0.3588879 | 0.3890351 |
| 10 | 0.17835563 | 0.3582911 | 0.4633533 |
| 11 | 0.21117954 | 0.3235916 | 0.4652289 |
| 12 | 0.20842624 | 0.2465875 | 0.5449862 |
| 13 | 0.27193372 | 0.3560535 | 0.3720128 |
| 14 | 0.19093504 | 0.3803897 | 0.4286753 |
| 15 | 0.18106451 | 0.3520488 | 0.4668867 |
| Mean across all folds | 0.191748 | 0.341987 | 0.466265 |

Supplementary Table 6. Average performance across folds for SuperLearner and base algorithms.

| Model | Mean AUC | SD AUC |
| --- | --- | --- |
| SuperLearner | 0.7713891 | 0.01460132 |
| Ranger | 0.7374766 | 0.02367066 |
| GLMNET | 0.7580662 | 0.01325249 |
| KSVM | 0.7573865 | 0.02056612 |

AUC: area under the curve; SD: standard deviation.

Feature importance was normalised, weighted by SuperLearner coefficients and aggregated across folds (Supplementary Table 7). These included features from several domains and ages. The most important predictors included lifetime parental financial stress, being male, being female (reference category gender diverse), religious involvement, and Parent 1 alcohol use problems.

Supplementary Table 7. Feature importance, normalised and weighted by SuperLearner coefficients and aggregated across folds for all 217 predictors included in the sensitivity analysis.

| **Feature** | **Weighted Mean** | **Weighted SD** |
| --- | --- | --- |
| Financial stress | 0.76032397 | 0.145022039 |
| Male | 0.717216439 | 0.099762296 |
| Female | 0.706967708 | 0.096776953 |
| Religious involvement | 0.566551126 | 0.104411476 |
| Parent 1 alcohol use problem | 0.561274946 | 0.127614123 |
| Pre-natal alcohol exposure | 0.544646528 | 0.099802785 |
| Moral peers (16) | 0.513340803 | 0.055650222 |
| Bully victimisation (16) | 0.51059031 | 0.068114068 |
| ADHD | 0.507897091 | 0.116866915 |
| Conduct problems | 0.449701054 | 0.093299416 |
| Extraversion (age 16) | 0.441458178 | 0.110487737 |
| Lower parental monitoring (14) | 0.413086043 | 0.069690972 |
| Moral peers (14) | 0.40238366 | 0.05935794 |
| Anxiety (12) | 0.401538527 | 0.077614084 |
| Housing insecurity | 0.391594934 | 0.081351897 |
| Neuroticism (16) | 0.388482354 | 0.087721418 |
| Anxiety (16) | 0.386782917 | 0.106219808 |
| Sleep problems (4) | 0.379514366 | 0.090834766 |
| Anxiety (14) | 0.37425931 | 0.063380993 |
| Delinquency (16) | 0.373162895 | 0.078651521 |
| Family SES (4) | 0.368718142 | 0.074469183 |
| Parent 1 self-efficacy (12) | 0.363188245 | 0.12795184 |
| Sleep quantity (12) | 0.34583758 | 0.083114773 |
| Parent 1 mental health disorder | 0.343105995 | 0.050492688 |
| Highest household qualification - university | 0.337347258 | 0.081001165 |
| Suicidal ideation (16) | 0.31556129 | 0.089344585 |
| Peer problems (14) | 0.313475795 | 0.081675316 |
| Consistent parenting (14) | 0.31259058 | 0.066426785 |
| Hyperactivity (14) | 0.289458294 | 0.074869795 |
| Emotional problems (16) | 0.288909319 | 0.088195413 |
| Parent 1 psychological distress (14) | 0.287398966 | 0.052130124 |
| Emotional problems (12) | 0.286455976 | 0.078610844 |
| Bully perpetration (16) | 0.286113034 | 0.043520858 |
| Parent support to child (14) | 0.284124383 | 0.048991393 |
| Area-level socioeconomic advantage and disadvantage (16) | 0.280808553 | 0.080314611 |
| Unsupervised time (14) | 0.279198782 | 0.112978014 |
| Unsupervised time (12) | 0.276939013 | 0.098181784 |
| Angry parenting (8) | 0.270534846 | 0.063813831 |
| Close to mum (12) | 0.265918461 | 0.096837137 |
| Parent 1 self-efficacy (14) | 0.264049944 | 0.052903772 |
| School belonging (14) | 0.260943669 | 0.053372672 |
| Housing insecurity (16) | 0.258787605 | 0.110591703 |
| Peer problems (16) | 0.257302458 | 0.062545682 |
| Hyperactivity (12) | 0.253969411 | 0.062875327 |
| Parent couple arguments (12) | 0.253455383 | 0.055616051 |
| Delinquency (14) | 0.252732918 | 0.032997786 |
| Parent 1 stressful life events (14) | 0.249133883 | 0.065653141 |
| Parent 1 stressful life events (12) | 0.248572223 | 0.072342812 |
| Transgender | 0.248011704 | 0.120624072 |
| Parent 1 stressful life events | 0.24621003 | 0.060460752 |
| Parent support to child (16) | 0.245583128 | 0.058728571 |
| Self-harm (14) | 0.241554056 | 0.082716138 |
| Parent 1 highest qualification | 0.241111983 | 0.064479309 |
| Peer communication (12) | 0.239832872 | 0.060861179 |
| Peer problems (10) | 0.238900025 | 0.036255256 |
| Parental warmth (6) | 0.235671459 | 0.046760041 |
| Parent couple arguments (6) | 0.235150274 | 0.076868711 |
| Lack of social support (10) | 0.232958556 | 0.096433073 |
| Close to mum (16) | 0.232758565 | 0.062687088 |
| Parent 1 stressful life events (4) | 0.232434019 | 0.048193021 |
| Self-harm ideation (14) | 0.231553464 | 0.050588434 |
| Early onset alcohol use | 0.231474622 | 0.070029363 |
| Parent relationship quality (4) | 0.230933868 | 0.055102847 |
| Peer trust (14) | 0.230686671 | 0.083670301 |
| Angry parenting (14) | 0.230517381 | 0.047522378 |
| Openness (16) | 0.230226665 | 0.070076277 |
| Consistent parenting (16) | 0.229193232 | 0.05863632 |
| Parent 1 difficulty of life (16) | 0.228095302 | 0.076586737 |
| Conduct problems (14) | 0.227482344 | 0.059546207 |
| Mum interest in child’s education (14) | 0.226167874 | 0.043364352 |
| Housing insecurity (14) | 0.225925137 | 0.111595802 |
| Home activities (4) | 0.224370473 | 0.047291901 |
| Overall happiness (16) | 0.224199546 | 0.046862136 |
| Parent 1 stressful life events (16) | 0.22404744 | 0.047983646 |
| Suicide attempt (16) | 0.223106749 | 0.076034446 |
| Highest household education | 0.222643938 | 0.064413881 |
| Sleep problems (10) | 0.222567839 | 0.046903221 |
| Parent 1 difficulty of life (14) | 0.222197564 | 0.064342487 |
| Hyperactivity (16) | 0.222051694 | 0.065236815 |
| Parent 1 psychological distress (16) | 0.221187285 | 0.09804834 |
| Parent 1 difficulty of life (12) | 0.220285409 | 0.042866793 |
| Suicide attempt (14) | 0.2178098 | 0.062174397 |
| Suicide plan (14) | 0.217021193 | 0.06989321 |
| Conscientiousness (16) | 0.21673626 | 0.047219284 |
| Overall happiness (14) | 0.216566934 | 0.077735931 |
| Parental warmth (10) | 0.216382278 | 0.070852328 |
| Consistent parenting (12) | 0.216336401 | 0.048058664 |
| Suicidal ideation (14) | 0.216014153 | 0.065319596 |
| Highest household education - diploma | 0.215995969 | 0.055900235 |
| Housing insecurity (12) | 0.21566427 | 0.067762865 |
| Bully victimisation (14) | 0.215342701 | 0.045267713 |
| Angry parenting (12) | 0.214420987 | 0.047777523 |
| Peer communication (14) | 0.213798713 | 0.041736625 |
| Overall happiness (12) | 0.213315127 | 0.053694643 |
| Consistent parenting (6) | 0.213308002 | 0.054180976 |
| Mum interest in child’s education (12) | 0.213055457 | 0.060898449 |
| Financial stress (12) | 0.212702208 | 0.045663973 |
| Parent 1 psychological distress (10) | 0.212370514 | 0.053589731 |
| Support available to parent (4) | 0.21193229 | 0.060407287 |
| Number of schools attended | 0.211742402 | 0.06770204 |
| Peer communication (16) | 0.211647244 | 0.037762964 |
| Peer problems (12) | 0.211512732 | 0.067713381 |
| Sleep problems (12) | 0.211443971 | 0.073133301 |
| Area-level socioeconomic advantage and disadvantage (12) | 0.211147622 | 0.055518368 |
| Discrimination (14) | 0.210849299 | 0.044652261 |
| Lack of social support (12) | 0.210837547 | 0.067675714 |
| Domestic violence (12) | 0.210382435 | 0.06755183 |
| Moral peers (12) | 0.21027453 | 0.051884024 |
| Emotional problems (10) | 0.210172669 | 0.059956324 |
| Angry parenting (10) | 0.210128065 | 0.040369385 |
| Discrimination (16) | 0.209690253 | 0.046937444 |
| Dad interest in child’s education (14) | 0.208820513 | 0.045940579 |
| Parent couple relationship quality (12) | 0.208035882 | 0.046066567 |
| Angry parenting (4) | 0.207790134 | 0.074282745 |
| Bully perpetration (14) | 0.207762365 | 0.049958125 |
| Conduct problems (10) | 0.207351156 | 0.049119112 |
| Out of home activities (4) | 0.206863582 | 0.051019785 |
| Financial stress (14) | 0.206578555 | 0.068076666 |
| Parental monitoring (12) | 0.206185959 | 0.06383636 |
| Dad interest in child’s education (12) | 0.206139176 | 0.056797839 |
| Out of home activities (6) | 0.206096313 | 0.060376288 |
| Depressive symptoms (12) | 0.205603348 | 0.039950945 |
| School belonging (16) | 0.204328006 | 0.042184969 |
| Peer trust (12) | 0.202686732 | 0.049424175 |
| Parent 1 Psychological Distress (4) | 0.2026568 | 0.051721352 |
| Financial stress (16) | 0.202313558 | 0.045752702 |
| Close to mum (14) | 0.202215393 | 0.052979424 |
| Parent support to child | 0.202132964 | 0.060986016 |
| Consistent parenting (8) | 0.20178426 | 0.073299741 |
| Home activities (10) | 0.200994288 | 0.0471058 |
| Domestic violence (4) | 0.199879527 | 0.045999591 |
| Parent 1 difficulty of life (6) | 0.197810031 | 0.047771603 |
| Parent 2 highest qualification | 0.197063143 | 0.045355043 |
| Parental warmth | 0.19656766 | 0.047265844 |
| Home activities (8) | 0.196095551 | 0.068370522 |
| Parent couple arguments (8) | 0.196079622 | 0.059071879 |
| Support available to parent (6) | 0.195830818 | 0.03598434 |
| Domestic violence (8) | 0.195394562 | 0.055554591 |
| Consistent parenting (10) | 0.19464907 | 0.043904898 |
| Parent 1 stressful life events (10) | 0.194357574 | 0.045880515 |
| Discrimination (12) | 0.193643522 | 0.046063039 |
| Domestic violence | 0.192586037 | 0.053325847 |
| Sleep quantity (16) | 0.19230149 | 0.04844501 |
| Sleep quantity (10) | 0.191746073 | 0.046073369 |
| Home activities (6) | 0.19172867 | 0.073886335 |
| Parent couple arguments (14) | 0.191607285 | 0.06140341 |
| Consistent parenting (4) | 0.191523679 | 0.05895691 |
| Neighbourhood liveability (14) | 0.191328239 | 0.037481977 |
| Parent 1 drug use disorder | 0.190679153 | 0.062723363 |
| Parent relationship quality (8) | 0.190644541 | 0.050862175 |
| Depressive symptoms (16) | 0.190428462 | 0.055851995 |
| Neighbourhood liveability (4) | 0.190302607 | 0.091501915 |
| School belonging (12) | 0.189883369 | 0.043084031 |
| Parental warmth (4) | 0.189215384 | 0.054464319 |
| Conduct problems (12) | 0.189096808 | 0.051780694 |
| Parental warmth (14) | 0.188086141 | 0.054274786 |
| Area-level socioeconomic advantage and disadvantage (14) | 0.187905718 | 0.044307242 |
| Neighbourhood liveability (10) | 0.187628766 | 0.045531302 |
| Close to dad (12) | 0.187336396 | 0.057246604 |
| Parent couple arguments (10) | 0.187304592 | 0.043404976 |
| Parent 1 self-efficacy (16) | 0.18642656 | 0.052121176 |
| Parent couple arguments (4) | 0.186381262 | 0.055794052 |
| Academic pressure from parents | 0.18637962 | 0.057489032 |
| Out of home activities (12) | 0.186184808 | 0.049207478 |
| Domestic violence (14) | 0.18532588 | 0.049634885 |
| Parent 1 Psychological Distress (12) | 0.185058677 | 0.057580881 |
| Close to dad (14) | 0.184888876 | 0.058887032 |
| Self harm (16) | 0.184002043 | 0.051920501 |
| Parent 1 difficulty of life (4) | 0.183935274 | 0.034838808 |
| Out of home activities (8) | 0.183929171 | 0.044166042 |
| Parent 1 difficulty of life (8) | 0.18346385 | 0.057757489 |
| Parent relationship quality (10) | 0.18345878 | 0.069156131 |
| Selfharm ideation (16) | 0.183233222 | 0.044837762 |
| Parent financial stress (10) | 0.182807225 | 0.054242787 |
| Delinquency (12) | 0.182715152 | 0.043959916 |
| Parent 1 self-efficacy (10) | 0.182431233 | 0.035005867 |
| Parent 1 self-efficacy (4) | 0.182222748 | 0.048166839 |
| Depressive symptoms (14) | 0.18105896 | 0.057587905 |
| Parent 1 stressful life events (8) | 0.180127275 | 0.050729999 |
| Parental monitoring (10) | 0.180100776 | 0.05579266 |
| Parent 1 Psychological Distress (8) | 0.179916872 | 0.040162084 |
| Number of homes lived in | 0.177488373 | 0.049459711 |
| Peer trust (16) | 0.176708756 | 0.052412852 |
| Discimination | 0.176675929 | 0.043376493 |
| Angry parenting (16) | 0.176563402 | 0.074075949 |
| Suicide plan (16) | 0.176528699 | 0.054597896 |
| Parent 1 Psychological Distress (6) | 0.175846452 | 0.052618836 |
| Parental monitoring (8) | 0.175547214 | 0.054456467 |
| Out of home activities (14) | 0.175464675 | 0.061248447 |
| Parental warmth (12) | 0.175413514 | 0.052774488 |
| Support available to parent (8) | 0.175412861 | 0.038302686 |
| Sleep quantity (14) | 0.175075924 | 0.048607849 |
| Support available to parent (12) | 0.17490061 | 0.056295963 |
| Agreeableness (16) | 0.174832381 | 0.056192109 |
| Area-level socioeconomic advantage and disadvantage (10) | 0.174514153 | 0.043603121 |
| Neighbourhood liveability (6) | 0.173486904 | 0.048761676 |
| Angry parenting (6) | 0.172283286 | 0.041439722 |
| Out of home activities (10) | 0.171548049 | 0.034245426 |
| Parent 1 self-efficacy (8) | 0.171109849 | 0.051963794 |
| Area-level socioeconomic advantage and disadvantage (4) | 0.170095474 | 0.04763582 |
| Parent 1 difficulty of life (10) | 0.169878025 | 0.037190331 |
| Sleep problems (6) | 0.169709525 | 0.040222952 |
| Parental separation (16) | 0.169468196 | 0.064038556 |
| Emotional problems (14) | 0.169260788 | 0.048037103 |
| Domestic violence (10) | 0.167623261 | 0.048631449 |
| Parental warmth (8) | 0.166727576 | 0.038767704 |
| Area-level socioeconomic advantage and disadvantage (6) | 0.165921065 | 0.049950592 |
| Domestic violence (6) | 0.165812928 | 0.037854573 |
| Sleep problems (8) | 0.164061909 | 0.045489257 |
| Parent relationship quality (14) | 0.16373697 | 0.039308867 |
| School achievement | 0.161605583 | 0.059370514 |
| Sleep problems (14) | 0.160966256 | 0.051738467 |
| Parent 1 self-efficacy (6) | 0.159928523 | 0.035033516 |
| Hyperactivity (10) | 0.156890451 | 0.056823805 |
| Parent 1 stressful life events (6) | 0.155853028 | 0.060780954 |
| Area-level socioeconomic advantage and disadvantage (8) | 0.1552339 | 0.04935435 |
| Neighbourhood liveability (8) | 0.151996538 | 0.057407497 |

**References**

1. Stone, A.L., et al., *Review of risk and protective factors of substance use and problem use in emerging adulthood.* Addictive Behaviors, 2012. **37**(7): p. 747-775.

2. Meque, I., et al., *Predictors of Alcohol Use Disorders Among Young Adults: A Systematic Review of Longitudinal Studies.* Alcohol and Alcoholism, 2019. **54**(3): p. 310-324.

3. Paterson, G. and A. Sanson, *The association of behavioural adjustment to temperament, parenting and family characteristics among 5‐year‐old children.* Social Development, 1999. **8**(3): p. 293-309.

4. Moffitt, T.E. and P.A. Silva, *Self-reported delinquency: Results from an instrument for New Zealand.* Australian & New Zealand Journal of Criminology, 1988. **21**(4): p. 227-240.

5. Bandara, D., Gasser, C., Jessup, K., Renda, J., Warren, D., Daraganova, G., *Factors associated with non-response in Growing Up in Australia: The Longitudinal Study of Australian Children*, in *Technical Paper No. 23*. 2020.
